# Understanding the Role of Support in Digital Mental Health Programs With Older Adults: Users’ Perspective and Mixed Methods Study

**DOI:** 10.2196/43192

**Published:** 2022-12-13

**Authors:** Judith Borghouts, Elizabeth V Eikey, Cinthia De Leon, Stephen M Schueller, Margaret Schneider, Nicole A Stadnick, Kai Zheng, Lorraine Wilson, Damaris Caro, Dana B Mukamel, Dara H Sorkin

**Affiliations:** 1 Department of Medicine University of California, Irvine Irvine, CA United States; 2 Herbert Wertheim School of Public Health and Human Longevity Science University of California, San Diego La Jolla, CA United States; 3 The Design Lab University of California, San Diego La Jolla, CA United States; 4 Department of Psychological Science University of California, Irvine Irvine, CA United States; 5 Department of Informatics University of California, Irvine Irvine, CA United States; 6 Department of Public Health University of California, Irvine Irvine, CA United States; 7 Department of Psychiatry University of California, San Diego La Jolla, CA United States; 8 Dissemination and Implementation Science Center Altman Clinical and Translational Research Institute University of California, San Diego La Jolla, CA United States; 9 Child and Adolescent Services Research Center San Diego, CA United States; 10 Department of Health and Human Services County of Marin San Rafael, CA United States

**Keywords:** older adults, mental health, digital mental health intervention, human support

## Abstract

**Background:**

Digital mental health interventions have the potential to increase mental health support among isolated older adults. However, the older adult population can experience several barriers to accessing and using digital health resources and may need extra support to experience its benefits.

**Objective:**

This paper aimed to understand what older adults experience as an important aspect of support during engagement in a digital mental health program. The program entailed 3 months of staff support to participate in digital literacy training and engage with the digital mental health platform myStrength, which offers support for a range of mental health challenges, including depression and anxiety.

**Methods:**

A total of 30 older adults participated in surveys and interviews to assess their experience of participating in a digital mental health program provided by county mental health services. As part of the program, participants attended 4 classes of digital literacy training, had access to the digital mental health platform myStrength for 2 months with staff support (and 10 months after the program without support), and received support from program staff during the entire 3-month program. Survey data were analyzed using descriptive statistics, and interview data were analyzed using thematic analysis.

**Results:**

A thematic analysis of the interview data revealed that participants valued ongoing support in 3 main areas: technical support to assist them in using technology, guided support to remind them to use myStrength and practice skills they had learned, and social support to enable them to connect with others through the program. Furthermore, participants reported that social connections was the most important aspect of the program and that they were mainly motivated to participate in the program because it was recommended to them by trusted others such as a community partner or because they believed it could potentially help others.

**Conclusions:**

Our findings can be used to inform the design of future digital mental health programs for older adults who may have unique support needs in terms of dedicated technical support and ongoing guided support to use technology and social support to increase social connectedness.

## Introduction

### Background

Mental health is an increasing concern among the older adult population. Even before the global COVID-19 pandemic, social isolation among older adults (ie, those older than 60 years) was a significant public health issue [1], and depression was estimated to be a more common health condition among older adults than dementia [2]. In addition to making it more difficult to access in-person care, the context of COVID-19 has raised concerns such as anxiety and loneliness [3]. According to a 2020 survey conducted by the American Association of Retired Persons Foundation and the United Health Foundation, the pandemic has caused an epidemic of loneliness and social isolation among the older adult population, with two-thirds of respondents saying that they experienced social isolation and 66% saying that their anxiety increased during the pandemic [4].

### Potential of Digital Mental Health Interventions

Digital mental health interventions (DMHIs) have the potential to remotely increase access to mental health support. Mental health support refers to any type of support aimed at protecting or promoting psychosocial well-being [5], such as therapy, education, and health promotion programs. Recent reviews have demonstrated the effectiveness of digital health interventions in reducing social isolation and depression in older adults [6-9]. However, older adults may experience several barriers to accessing and using digital health resources, such as lack of knowledge and awareness of digital health resources, little exposure to and experience with technology in general, and lack of confidence in using a computer [10]. Rather than increasing access to care, these barriers to digital health access may instead result in larger health care inequalities [11-13].

To better understand older adults’ perspectives toward the concept of mental health technologies, the study by Andrews et al [14] held a discussion group with older adults and enabled them to interact with various mental health apps and websites during a session to increase awareness. The authors found that there were overall positive attitudes toward mental health technologies. However, although study participants were aware of mental health websites, awareness of these websites did not motivate their actual use [14].

### Implementation of DMHIs for the Older Adult Population

Exposure and awareness alone appear to be insufficient for older adults to engage with DMHIs. A recent report by the Substance Abuse Mental Health Services Administration highlighted the need for support and digital training when offering DMHIs to older adults [15]. It is well known that guided DMHIs (eg, in the form of an internet-based or remote therapist or coach) generally have higher engagement than self-guided interventions [16], and this may be especially true for the older adult population. For example, Egede [17] found that web-based cognitive behavioral therapy was effective in improving depression and anxiety symptoms among older adults, but this was applicable only when a therapist was present to provide guidance. If an individual coach is not available, peer support can also be an effective and cost-effective type of support for web-based cognitive behavioral therapy interventions for older adults [18].

### Research Question

Prior recommendations to provide support have been mainly evaluated from an operational (eg, staffing considerations) perspective, with the aim of facilitating successful implementation of an intervention and achieving desired outcomes, rather than from a user’s perspective. For example, several recent studies have focused on the effectiveness of DMHIs with older adult participants and have provided support to older adults in the form of therapists, health professionals, research assistants, and coaches [3,17,19]. Support improves the effectiveness of DMHIs, and positive and concrete support messages have been associated with better clinical mental health outcomes [20]. However, few studies have assessed how and whether different types of support affect older adults’ user experiences. There are different types of possible support [21,22] that may be more or less suitable depending on the type of barriers they are supposed to address. For example, although text-based reminders may be suitable for people who tend to forget to use an intervention [22], they may not be sufficient to address a lack of motivation, social isolation, or low digital literacy (ie, a person’s ability to find, evaluate, and communicate information through digital platforms).

It is important to consider not only what types of support older adults need but also what they value, as participants’ experiences affect real-world use and can be crucial in whether they would continue to engage with a digital intervention beyond a research setting.

This paper reports on a study situated within a digital mental health program with older adults. The aim of this study was to evaluate older adults’ experiences with the program and specify what they experienced as important aspects of support while they were taking part in the program. The research question we aimed to address was as follows: What do older adults experience as important factors of support during engagement in a digital mental health program?

## Methods

### Overview

Thirty older adults participated in a digital mental health program for 3 months. The aim of the program was to engage English-speaking and Spanish-speaking isolated older adults with technology and to improve their well-being and sense of social connectedness. Participants experienced several types of isolation. First, everyone experienced some degree of social isolation as the program took place during the global COVID-19 pandemic. Second, older adults were selected based on geographic isolation, and finally, Spanish-speaking participants were also selected based on perceived isolation by program staff due to language, culture, and generational barriers.

Participants received digital literacy training as part of the program. Participants were given free access to the digital mental health platform myStrength for 1 year, and those who did not have the required resources to use myStrength were provided with tablets and an internet connection.

### myStrength Description

Participants were given 1-year access to myStrength, a digital and mobile-based mental health platform [23]. myStrength was chosen as the technology based on feedback from an exploratory study of multiple mental health apps with older adults in Marin County. The platform offers evidence-based resources to provide support for a range of mental health challenges, including depression, anxiety, stress, chronic pain, drug or alcohol recovery, and insomnia. It offers tools such as dialectical and cognitive behavioral therapy courses, acceptance and commitment therapy materials, mood and sleep tracking, short videos teaching mindfulness, community forums, motivational quotes, spiritual resources, activities ranging from 2 to 10 minutes, and a library of more than 1600 mental health resources. Participants can select user interests when signing up and receive personalized recommendations aligned with these interests. The participants could access any of the resources as many times as they wanted.

During the program, participants received weekly phone calls from the program staff to check in and to encourage them to use the platform. In addition, participants received at least one in-person visit from nurses to help them access and use technology. myStrength is offered in both English and Spanish. The Spanish version offered some content such as videos in Spanish, but the platform was not fully translated into Spanish (eg, the menu to navigate to content remaining in English).

### Study Design and Procedure

To address our research question, we conducted a mixed method study, in which participants took part in 3 surveys and an interview during the program, and 1 follow-up survey 8 months after the program (the survey instruments and interview protocol are included in Multimedia Appendix 1). The purpose of the surveys was to assess participants’ satisfaction with the digital literacy training, myStrength, and the program overall and to measure any improvement in outcomes throughout the program. The purpose of the interviews was to gain a more in-depth understanding of participants’ experiences with the program and the support provided, as well as any barriers they experienced in participating.

Older adults were invited to participate in a digital mental health program initiated by Marin County Behavioral Health and Recovery Services. Marin County is located in Northern California; its largest ethnic groups are White (non-Hispanic) and Hispanic, with a population of approximately 259,000 people, of which approximately 22% are aged ≥65 years [24]. The program was part of the Help@Hand project, a state-wide evaluation project in California that aims to understand how digital therapeutics fit within the public mental health care system. The specific aim of Marin County’s program was to engage isolated older adults (both English- and Spanish-speaking older adults) with technology and to enhance their well-being and sense of social connectedness. Program participants were offered a series of 4 digital literacy classes to enhance their technology skills and were then given free access to the digital mental health platform, myStrength. The program took place from March to June 2021 and consisted of five phases:

1. Phase 1: Onboarding participants by giving them access to required resources, such as a device and an internet connection, to be able to participate in the program (4 weeks)
2. Phase 2: Providing digital literacy training consisting of four 2-hour web-based classes to establish or improve participants’ digital literacy skills (4 weeks)
3. Phase 3: Providing participants free access to the myStrength platform and nurse promotores support (8 weeks)
4. Phase 4: Debriefing participants at the end of the program and conducting surveys and interviews (8 weeks)
5. Phase 5: Providing continued free access to myStrength after completion of the program (10 months)

When referring to the program, we refer to phases 1 to 4 as the 3-month period during which staff support was provided to the participants.

**Figure 1:**
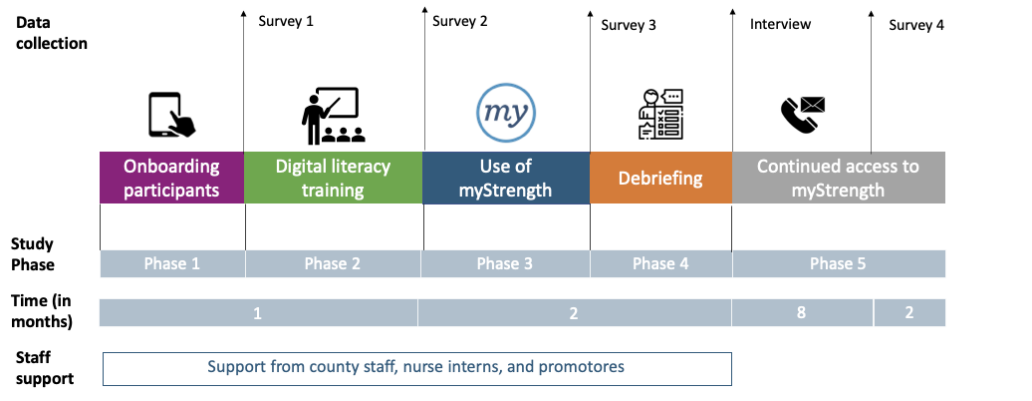
Study timeline, showing the different study phases and data collection points.

There were 5 key data collection points (Figure 1): 1 survey at the beginning of the program (phase 1), 1 survey after the digital literacy training and before myStrength was made available (between phases 2 and 3), 1 survey and 1 semistructured interview at the end of older adults’ participation in the program (phase 4), and 1 follow-up survey 8 months after the end of the program (phase 5). The first 3 surveys were completed on the web or over the phone, and interviews and follow-up surveys were conducted over the phone. For each data collection point, the research staff contacted participants via phone to remind them to complete the survey or interview for a maximum of five attempted phone calls. Participants could complete the surveys and interviews in either English or Spanish.

The data collection instruments were developed in English. The first 3 surveys and the interview guide were translated into Spanish by an external translation vendor. These translations were reviewed for accuracy and readability by 3 Spanish-speaking members of the research team. For example, vendor translation sometimes used formal words that are not typically used in everyday language, or the intent of a question was lost. These translations were updated to better encapsulate the original intent of the questions. The fourth follow-up survey was translated directly by the Spanish-speaking members of the research team.

Digital literacy training is offered in both English and Spanish. The training consisted of four classes on the following topics: (1) computer basics, (2) internet basics, (3) email basics, and (4) myStrength. All digital literacy classes were voluntary for both participants and program staff.

Because of the study taking place during the COVID-19 pandemic, digital literacy classes had to take place remotely over Zoom videoconferencing. However, because the participant population had little prior technology experience, Marin County decided to provide tablets and internet connections for those participants who needed it, and each participant received at least one in-person visit by the program staff to ensure that all participants were able to access the web-based training and engage in the program. To support participants throughout the program, Marin County partnered with volunteer nurse interns from 2 local universities and *promotores* (volunteers from the North Marin Community Service’s Promotores Program that work closely with the Spanish-speaking community in Marin County). Marin County chose to partner with nurse interns and promotores because they were trained and experienced community partners; furthermore, promotores were trusted community leaders in the Spanish-speaking community. The promotores network works to bridge and connect the community with health services and community engagement (for more information about the program, see the North Marin website [25]). The support provided by the interns and promoters included the following:

- Technical support, such as helping participants get connected to Wi-Fi,
- support during digital literacy training, such as teaching participants how to use their devices and Zoom app to participate in digital literacy classes,
- checking in with participants about their participation in the program, and
- connecting participants to resources and mental health services.

Participants were also offered the option of having a staff member visit them in person or provide remote support. Support was ongoing throughout the program, with weekly check-in calls from staff, nurse interns and promotores, and in-person visits as needed for participants who experienced more challenges with technology. The number of visits ranged from 1 (all participants had at least one in-person visit) to 10 visits.

### Recruitment

To help recruit older adult participants for the program, the county approached existing community-based organizations and agencies working with isolated adults, such as West Marin Senior Services, Meals on Wheels, and organizations that work closely with the Spanish-speaking older adult community. They also approached divisions in the county that already organized health programs for older adults, as they had the infrastructure and relationships in place to reach the older adult population.

Recruitment was primarily done by sharing information about the program in partnership with West Marin Senior Services and the Telehealth Equity Project in the Marin County Division of Aging. In addition, information was shared by the promotores within their network to recruit Spanish-speaking older adults*.* One of the county staff members was a *promotora* who had full access to the network. These partners also helped identify isolated older adults.

The program was specifically intended for isolated English- and Spanish-speaking older adults, as they were identified by county staff as underserved populations in the county. Participants expressed interest in the program by completing a screener form to assess their eligibility (Multimedia Appendix 2). This form can be completed on the web or over the phone in English or Spanish. Participants had to be 60 years or older (the threshold age for older adults as defined by the United Nations [26]) and be able to speak, read, and write in either English or Spanish. If participants had any mental or physical challenges that could limit their participation in the program, such as hearing or vision loss, they were contacted by a staff member to determine their eligibility. Program participants were expected to participate in surveys and interviews as part of the program. Marin County aimed to recruit 15 English- and 15 Spanish-speaking isolated older adults for the program, motivated by perceived capacity and the number of people the county staff were able to handle overall and recruit.

### Participants

A total of 30 participants were enrolled in the study. The first survey (phase 1) was completed by 28 participants, the second survey (between phases 2 and 3) was completed by 25 participants, the third survey (phase 4) was completed by 23 participants, the fourth survey (after phase 4) was completed by 18 participants, and 24 participants participated in the interview.

One participant did not complete the demographic survey. Among the 29 participants who provided demographic data, their ages ranged from 60 to 89 years, with a mean age of 72 (SD 7.8) years, and 27 (93%) participants identified as female. Furthermore, 28% (8/29) identified as non-Hispanic White, 24% (7/29) as Mexican or Mexican American, 17% (5/29) as Central American, 10% (3/29) as South American, 10% (3/29) as other White, 3% (1/29) as other Hispanic or Latinx, 3% (1/29) as Black or African American, and 3% (1/29) as other or African. In addition, 48% (14/29) of participants indicated that English was their preferred language, and 45% (13/29) of participants reported Spanish as their preferred language. One participant indicated a preference for Vietnamese, and 1 participant did not indicate a preference. One Spanish-speaking participant did not have reading proficiency and completed the survey verbally with staff assistance.

### Measures

#### Overview

Table 1 presents an overview of the study measures and the points at which the measures were assessed. Next, we describe these measures in detail.

**Table 1:** The measures of the study^a^.

Study measures	Survey 1 (phase 1)	Survey 2 (between phase 2 and 3)	Survey 3 (phase 4)	Interview (phase 4)	Survey 4 (phase 5)
Important aspects about technology		✓		✓	
Activities people would like to do using (mental health) technology		✓		✓	
Experience with myStrength			✓	✓	✓
Use of myStrength			✓	✓	✓
Other resources and strategies used to support mental health and well-being				✓	
Overall experience with or impact of the program			✓	✓	✓
Experience with program staff				✓	
(If applicable) Reasons for dropping out of the program early				✓	
(If applicable) Reasons for nonuse of myStrength			✓	✓	✓
Digital literacy, mental health concerns	✓				
Loneliness and social isolation	✓	✓	✓		
Other health outcomes (eg, mental health improvements, and stigma reduction)				✓	

^a^The columns indicate at which data collection points the measures were assessed.

#### Digital Literacy

At the start of the program (phase 1), participants were asked to rate 2 statements related to digital literacy (eg, “I am confident using technology to look up information”) taken from the Mental Health Literacy Scale [27]. They were asked to rate these statements on a Likert scale ranging from 1 (strongly disagree) to 5 (strongly agree).

Participants were also asked about their technology use on the screener form. The purpose of these questions was to assess the extent to which support was needed for the onboarding participants.

#### Loneliness

Loneliness was measured at 3 time points: at the beginning of the program (phase 1), after digital literacy training (phase 2), and at the end of the program (phase 4). Loneliness was measured using the Three-Item Loneliness Scale [28], a shortened version of the University of California, Los Angeles Loneliness Scale [29]. Participants were asked to rate 3 statements related to loneliness on a 3-point Likert scale ranging from hardly ever (1) to often (3; eg, “How often do you feel left out?”) with a total added score ranging from 3 to 9. People with a score of 6 or higher were grouped as lonely [30].

#### Social Isolation

Social isolation was measured at three time points: at the beginning of the program (phase 1), after digital literacy training (phase 2), and at the end of the program (phase 4). The social interaction subscale of the Modified Duke Social Support Index [31,32] was used to measure social isolation. The 6-item scale asked participants about the frequency of social interactions in the past week (eg, ‘How many times in the past week did you talk with friends or relatives on the telephone?’) and how many people in their local area they could depend on. The total score ranges from 0 to 17. Individuals were considered socially isolated if they scored 6 points or less [30,33].

#### Mental Health

At the beginning of the program (phase 1), participants were asked a multiple-choice question on whether they had ever experienced or were currently experiencing any mental health challenges. Participants indicated that they had not experienced any challenges, had been diagnosed with a mental health condition, had not been diagnosed but were experiencing challenges, or could self-describe their experience. The survey explained that many different terms could be used to refer to these experiences, including mental health challenges, emotional distress, and psychological disorders.

#### Important Aspects of Mental Health Apps and Activities People Would Like to Do Using (Mental Health) Technology

Before engaging with myStrength (before phase 3), participants were asked what they considered important aspects of mental health technologies and what they would like to do using technology. For each of these questions, they were instructed to “select all that apply” from a list of 9 and 8 options, respectively, or give an answer in their own words. These lists were based on common needs from the literature regarding mental health technology [16], as well as common answers given by community members in prior studies of the Help@Hand project. Participants were also asked to elaborate on the important aspects and any barriers to use during the interviews.

#### User Experience of myStrength

After engaging with myStrength (after phase 3), participants were asked about their experience using myStrength (eg, their satisfaction using myStrength, its perceived usefulness, ease of use, and cultural competency). Participants were asked to rate 15 statements (eg, “I think myStrength is easy to use”) on a 5-point Likert scale ranging from strongly disagree (1) to strongly agree (5). The survey items were based on the Unified Theory of Acceptance and Use of Technology questionnaire [34], which is used to evaluate technology acceptance and adoption.

#### Continued Use and Experience of myStrength

Participants were contacted for a follow-up survey 8 months after the program was concluded (phase 5). The aim of the final survey was to learn more about participants’ continued use and experience of myStrength after the end of the program and staff support and asked questions similar to survey 3 (phase 4) applied to the period after the staff support had ended. Survey questions assessed people’s self-reported use of, and intention to use, myStrength, reasons for use and nonuse, challenges using myStrength, people’s perception of myStrength, facilitating conditions that would make it easier to use myStrength, and the overall impact of the program.

#### Interviews

Participants were invited for an interview at the end of their participation in the program (phase 4). The aim of the interviews was to gain a more in-depth understanding of participants’ experiences with the program. The research team developed an interview guide with discussions and sample questions. The topics covered in the interview guide included the following:

1. Overall experience with the program
2. Health outcomes (eg, mental health improvements and stigma reduction)
3. Experience with program staff
4. Other resources and strategies used to support mental health and well-being
5. If applicable, reasons for dropping out of the program early

### Ethics Approval

The study was approved by the University of California, Irvine, Institutional Review Board (review number 20195406).

### Participant Consent and Compensation

Before each survey and interview, the participants were emailed a study information sheet that was reviewed and approved by the Institutional Review Board. For interviews and surveys over the phone, the sheet was reviewed at the start of the phone call with an opportunity for participants to ask questions. Participants were asked for their permission to audio-record the interviews at the start of the phone call; participants were still able to participate if they declined to be recorded. In addition, participants were asked to sign a participation agreement and a device user agreement by Marin County at the start of the program. The participation and device user agreements were reviewed and approved by the county’s legal department. All collected research data were stored securely and confidentially on a password-protected secure server. Participants received a US $10 gift card for each survey that they completed and a US $20 gift card for completing the interview. The survey took about 20 minutes to complete, and the interview took approximately 40 minutes.

### Data Analysis

We analyzed the survey data using descriptive statistics, such as frequency counts. The analyses were performed using SAS statistical software (version 9.4; SAS Institute Inc) [35]. Changes in loneliness and social isolation before and after the program were assessed using paired 2-tailed *t* tests.

Audio recordings of the interviews were transcribed verbatim in the language they were conducted in. The transcripts were analyzed by 2 members of the research team (one of whom was fluent in both English and Spanish) using thematic analysis. The themes were derived as follows: the interview protocol was developed to ascertain the overall experience with the program based on some collectively agreed upon outcomes among the research team, the county, and the broader collaboration that supported the Help@Hand Project; thus, these constructs (eg, myStrength user experience and mental health improvements) were used as a starting point for the thematic analysis, and interview questions were grouped based on construct (eg, all questions related to barriers to use of myStrength were grouped together). However, the interview also allowed for emergent ideas either not mapped directly to the outcomes of the project or high enough that important subthemes could emerge (eg, in the case of myStrength use). Transcripts were first independently reviewed by each research member. The 2 members then met to discuss patterns and notes and iteratively developed a codebook that fit within the topic categories and captured the patterns identified in both English and Spanish transcripts. Spanish transcripts were then translated into English by an external translation vendor, and transcripts were coded by 1 researcher and discussed with the other research team member. The desktop version of the qualitative analysis platform Dedoose [36] was used to code the transcripts.

## Results

### Overview

The following section presents an overview of the survey and interview results. The results are organized by theme rather than by method (ie, survey vs interview). Themes were based on interview themes (eg, all findings related to myStrength user experience were grouped together). Throughout this section, we report the number of people who provided a particular survey answer and the total number of people who responded to the survey question.

### Motivation to Participate in the Program

Participants reported that they became aware of the program primarily through word of mouth and recommendations from others. The reasons for deciding to participate were as follows: (1) participants trusted the program based on recommendations from others who had participated in other programs offered by Marin County, (2) they wanted to contribute to a program that may help other people, and (3) they were interested in learning something new.

Before engaging with myStrength, participants, overall, reported positive expectations about myStrength on the survey: they believed that it could be useful in their everyday life (18/26, 69%) and improve their mental health (15/16, 94%), and around half (14/26, 54%) of them believed that it might meet their mental health needs. However, during the interviews at the end of the program, it was revealed that not everyone knew beforehand what the app did or had a clear image of what “digital mental health” was: “I had no idea what it was about, so I had no expectations. I didn’t know about it. She told me about it, and so it’s hard to have expectations on something you don’t know about.”

### Support During the Program

#### Technical Support

Although participants were excited about participating in the program, they had varying degrees of readiness to begin the program. For example, before starting the program, 48% (11/23) of participants reported that they did not have the required devices to use myStrength and participate in the digital literacy classes; these participants were given a tablet by Marin County to participate in the program. In addition, 21% (6/28) of participants needed support to access Wi-Fi, and 21% (6/28) of participants said they had never accessed the internet before. The technical support available through the program was experienced by participants as very useful, as expressed during the interviews: “The one-on-one technical [support]...[opened the door to new technology].”

#### Support to Improve Digital Literacy

Participants indicated different levels of confidence in using technology in the surveys, and some not only needed support to access technology but also needed support to learn how to use technology. Almost half (12/26, 46%) of the participants reported that they were not confident about using technology at the start of the program. Furthermore, 76% (19/25) of participants did not know how to download a mobile app on their phone, and 62% (16/26) of them reported they were not comfortable using technology to support their well-being: “Todo era nuevo para mí. No sabía manejar estas cosas. Simplemente, el celular... (Everything was new to me. I didn’t know how to handle these things. Simply, the cell phone...)”

However, participants were interested in learning new technology skills and improving their digital literacy. Specifically, participants were keen to learn how to use smartphones (24/26, 92%), the internet (24/26, 92%), computers (22/26, 85%), and how to use technology to support their mental health (22/26, 85%).

#### Guided Support

Overall, 80% (24/30) of participants participated in at least one of 4 digital literacy classes. Even though participants had positive experiences with digital literacy training and gained digital skills, not everyone felt confident enough after the training to use web-based tools on their own for mental health. Participants reported feeling overwhelmed by the digital literacy training at times and the variety of options they were exposed to and noted the challenges of trying to teach and accommodate different needs in a group setting:

Well, for a long time, I found it quite confusing. And somehow I guess I was feeling negative at first and thinking, Oh, dear...It’s sort of a new speak, you know? [laughs]...But later, I began to see some of the value in it [digital literacy training] and realized it wasn’t really the way I was feeling it would be...Well, it’s certainly helped to be able to be in touch with family. And so, that was lovely.

To apply the skills they learned, participants not only needed human support at the beginning of the program but also continued support during the program. This ongoing support was experienced as helpful throughout the program:

Oh, I thought it was great. The nurse—Yes, she was wonderful. She just went overboard to do the training as best she could...Oh, yes, they were very willing to do whatever I needed help with...She was continually there. And if I needed her to help me with something, she went out of her way to come over and help me at that particular time...[helped] in person...Yes, exactly. If I needed help, I need someone right here.

A total of 5 participants reported that they did not have the support of family and friends to remind them or help out if they had any technical difficulties, and staff, therefore, checked in with participants one on one throughout the program. This personal support was experienced as very helpful: “The one-on-one contact was very good, excellent.”

### Social Component

#### Using Technology to Connect to Others

Social connection was an important aspect of the program for participants: 75% (21/28) of participants reported that they wanted to learn how to use technology to connect with friends and family. This was echoed during the interviews as follows:

I think it’s WhatsApp or something like that?...Yeah, they, my friends have been telling me, so that way you don’t have to pay long distance phone calls...So that’s what I remember. So I think, you know, it’s good to have that.

#### Impact of Program on Loneliness and Social Connectedness

A total of 38% (11/29) of participants reported experiencing mental health challenges at the beginning of the program. During the interviews, participants described how the global coronavirus pandemic had affected them, including increased isolation, lack of social connection and activities (including evolving practices, tensions in personal safety and risk vs social connection and mental health), safety measures, and even some having experienced the virus. In addition, many participants reported feeling isolated or lonely. For some, health conditions impacted feelings of isolation:

Sí, ahorita con la pandemia, sí me siento sola. Y como que sí me da miedo quedarme sola porque, primero, hay tantas cosas que me han pasado... (Yes, right now with the pandemic, I do feel alone. And yes I’m afraid of being left alone because, firstly, there are so many things that have happened to me...)

Overall, 78% (18/23) of participants indicated on the survey at the end of the program that they felt more connected to others because of the program. To compare any changes in loneliness and social isolation during the program, we considered 22 participants who answered the survey questions on loneliness and social isolation at baseline and at the end of the program. There was a statistically significant decrease in loneliness from the beginning of the program (mean 6.6, SD 1.7) to the end (mean 5.5, SD 2.1; t_21_=3.04; *P*=.006). A total of 17 participants scored high on loneliness at the beginning of the program; at the end of the program, only 9 participants scored high on loneliness.

There was also a statistically significant decrease in social isolation from the beginning of the program (mean 7.4, SD 4.0) to the end (mean 9.2, SD 5.0; t_21_=−3.11; *P*=.005). A total of 7 participants were considered socially isolated at the beginning of the program and 4 were considered socially isolated at the end of the program.

Participants expanded on feelings of connection during the interviews. For example, interacting with other participants during digital literacy classes helped with loneliness, and it improved feelings of connectedness and purpose:

Yes, it [program] did help [impact feelings of connectedness]...Well, it just—I took some classes on Zoom, and going through the program, myStrength, yeah, it broadened my atmosphere a little bit, a lot I can say...

Y esto me ayuda porque ya me distrae, es algo como que puedo hacer. Porque no tengo ganas de nada, ni de limpiar la casa ni de...Ahorita no tenía ganas pero de nada. Y con esto sí me ha ayudado mucho. (And this helps me because it distracts me, it’s like something I can do. Because I have no desire for anything, not to clean the house or...Right now I did not feel like doing anything. And with this it has helped me a lot.)

Participants also felt connected because they learned how to use technology to reach out to family and friends during the digital literacy classes: “I was pretty much at home most of the time and alone, so that was nice to be able to get into the technology, and reach out to more people*.*” Participants were satisfied with the program and hoped that more programs like this would be offered in the future.

### Digital Literacy Training Experience

Most (19/24, 79%) participants were satisfied with the digital literacy training, and participants reported various benefits from participating in the training; they (13/17, 76%) reported that they were more likely to use technology and felt more socially connected (12/19, 63%), and the percentage of participants who felt confident using technology increased from 31% (8/26) before training to 73% (19/26) after training.

### myStrength Use and Experience

Overall, participants had a positive experience with myStrength: they recommended myStrength (18/23, 78%), found it useful (17/23, 74%), and found it easy to use (15/23, 65%). Benefits of myStrength were that it changed how they thought about mental health and helped them recognize symptoms:

Maybe it made me more aware so that when I got in this bad mood a couple weeks ago, that...I didn’t know. [chuckles] One of the sections of myStrength was—I think it was Depression...And I’ve never had depression. So, I don’t know what it’s like. But I think it was last week, all of a sudden for about two days, I think I felt what might be depression...And so, I mean to go back and check that out.

In addition, 70% (16/23) of participants indicated that they had used myStrength for 2 months at the end of the program, and 52% (12/23) had said they used it several times a week or daily. Furthermore, 57% (13/23) participants self-reported that they had stopped using myStrength at some point during the study. The main reasons participants mentioned for not using myStrength were that they had no time (4/13, 31%), health reasons such as chronic fatigue (4/13, 31%), technical issues (3/13, 23%), and they did not think they needed it (2/13, 15%): “No funciona mucho, falla el Internet. Por eso a veces no lo ocupo. (It doesn’t work much, the Internet fails. That’s why sometimes I don’t use it.)”

Although (18/23, 78%) participants agreed that myStrength valued cultural differences, several Spanish-speaking participants mentioned that many aspects of the Spanish version were available only in English:

Pero cuando yo entré a myStrength, todo es en inglés...porque, como te digo, si sería en español, entonces iría más rápido al video...Ahorita estoy como poquito a poquito...en la tablet, son en español las instrucciones. Pero cuando voy a myStrength, todo sale en inglés...lo que yo digo de ellos es que si estuviera en español el programa, la mayoría de los programas, o sea, en inglés y en español, sería maravilloso. (But when I entered myStrength, everything is in English...because, as I told you, if it would be in Spanish, then I would go faster to the video...Right now I’m like little by little...on the tablet, the instructions are in Spanish. But when I go to myStrength, everything comes out in English...what I say about them is that if the program were in Spanish, most of the programs, that is, in English and Spanish, it would be wonderful.)

When thinking about mental health technology beyond myStrength, important aspects for participants were that the data should be kept private (20/28, 71%), the app would be free (20/28, 71%), and the app would not have a negative effect on their device, such as draining their battery (19/28, 68%).

### Continued Use of myStrength: 8-Month Follow-up

Eight months after the program and staff support ended, 16 (53%) of the 30 participants completed the follow-up survey. During the program, participants overall had a positive experience with myStrength, reported high use, and reported that they were more likely to use technology to support their well-being; only 1 participant reported that they were still using myStrength at the time of the follow-up survey, even though participants still had free access to the platform. Furthermore, 75% (12/16) of the participants reported that they experienced technical issues that prevented them from continuing to use myStrength. Technical issues included forgetting how to use a tablet, smartphone, or computer and not knowing how to log back into the platform after being logged out. In addition, 19% (3/16) of the participants also reported significant life events, such as illness or injury, contributing to nonuse. Although 50% (8/16) participants reported that they intended to use myStrength again in the future, 44% (7/16) of participants also indicated that ongoing support would make it much easier to use myStrength.

## Discussion

### Principal Findings

This paper sought to share what older adults consider important factors of support when engaging in a digital mental health program. We found that participants valued different types of support to stay engaged in the program: technical support, guided support to be reminded to engage with the program, and social support. Social connections were the most important aspect of the program for participants. In addition, even though participants were excited to participate in the program, they did not have a clear image of digital mental health and were mostly motivated to participate in the program because it was recommended by someone they trusted, such as a community partner with whom they were familiar or peers who had participated in similar programs offered by the county. Next, we discuss these findings in more detail and the implications they may have for the future design of digital mental health programs for older adults.

### Technical Support and Guidance

Although the program showed high engagement and an improvement in loneliness and social connectedness, it is important to understand these results in the context of considerable hands-on staff support, rather than solely attributing these improvements to mental health technology. When contacted 8 months after the program—and thus the support—had ended, most respondents had stopped using myStrength.

Nonuse of a digital health app does not always have to be negative [37]; for example, users’ needs may have changed, or they may have received sufficient benefits from the app and no longer need it. Therefore, it is important to look beyond user engagement and understand the reasons behind the potential nonuse of a DMHI. In our study, participants indicated that they intended to use myStrength beyond the program but experienced several barriers in doing so. For example, in the follow-up survey, respondents noted that they encountered technical issues and were unable to easily overcome them without any technical assistance. This is consistent with previous work that found technical issues to be a common barrier to engagement with DMHIs [16], and technical assistance has been shown to be an important aspect in facilitating engagement [38,39].

Providing continued support from a trained person was shown to be important in reminding older adults to use the technology and troubleshoot in case of technical issues during the program. Furthermore, it was important to provide ongoing personal support. In a separate publication [40], we discuss the role of support in the program from a staff perspective, who confirmed that, in particular, participants with lower digital literacy needed considerable support and reminders in between classes to practice what they had learned during the digital literacy training and to use the technology.

These findings indicate that when integrating DMHIs into existing programs, typical measures of success, such as efficacy or changes in mental health outcomes, may not be sufficient to achieve a long-term beneficial impact. The real-world success of DMHIs in older adults may depend on the presence of intensive and ongoing support. In addition, this support may have benefits for both sustaining engagement and alleviating social isolation.

To ensure that this resource-rich support is sustainable, it is important to assess support needs and build wrap-around services around DMHIs for older adult populations. For example, there may be an opportunity to build and maintain close partnerships with local agencies that already work closely with older adult communities, such as home nursing services.

The facilitating factor of personal guidance is widely known in the digital mental health literature, and guided DMHIs typically have higher engagement than unguided DMHIs. For example, 2 studies that evaluated a self-guided DMHI found that participants regularly forgot to use the intervention [41,42]. In contrast, studies that compared versions of an intervention with and without guidance (in the form of emails, phone calls, or text reminders by a human coach) found that guided use increased user engagement [43-45]. In addition, prior studies found that participants preferred whether an intervention was integrated into therapy or an existing program rather than being offered as a standalone tool [43,46,47].

However, human support is resource intensive and may not always be feasible. Therefore, different types of support may be used to address the different types of challenges people experience. For example, for older adults with higher digital literacy, email and text reminders could remind people of the technology at regular intervals; automated reminders have been found to facilitate engagement with the general population and have been experienced positively [16]. In addition, a larger digital literacy initiative may be needed to improve skills in overcoming technical issues, such as formal training classes or more informal walk-in sessions to troubleshoot common technical issues. Digital literacy training in this study increased participants’ confidence in using technology and the likelihood of using technology and may further increase their ability to engage in digital programs. In addition, these classes can provide an opportunity to learn how to use technology beyond a mental health tool, such as communication technologies, to connect with friends and family, which can impact social connectedness. Individual human support can be dedicated to those needing more personalized support or motivating encouragement.

### Social Connectedness Beyond DMHIs

Both feelings of loneliness and feelings of being socially isolated significantly decreased from before to after the program. This decrease may indicate that participation in the program reduced social isolation in some participants. Participants indicated that they liked being able to connect with people such as other participants, digital training teachers, county staff, nurses, and promotores during the program, and some even took part in the program because they wanted to help others in improving the program for future deployment among older adults.

This is in line with prior research highlighting that offering social opportunities is an important facilitator of DMHI use among older adults [48]. For example, a study evaluating a physical activity program with older adults offered participants the option to complete activities in a group setting with other participants [48]. Those who chose to complete activities as a group reported greater social connectedness and improvements in their mental health, compared with those who chose to do the activities on their own.

Given the great value placed on a social component, previous work has recommended that DMHIs should incorporate a feature or ability to connect with others [49]. However, although our study participants did consider a social component to be important when considering mental health technologies, these interactions may not necessarily have to be through the DMHI: participants in our study also valued being able to connect with others through the program and the digital literacy classes by connecting with staff members and loved ones by learning communication technologies such as WhatsApp and Zoom. This indicates that different types of social connections may be important: connections with other community members who may or may not have shared mental health experiences, connections with family and friends, and connections with supporting staff.

Future digital mental health programs could host sessions where participants may be able to meet others taking part in the program and connect with others and may focus their digital literacy initiatives on teaching skills on how to connect and communicate with loved ones.

### Endorsement and Outreach by Community Partners

Our study also revealed the importance of collaborating with community partners who have a close relationship with the community to get a digital mental health program that starts with older adults. Participants primarily indicated that they became aware through word of mouth and decided to participate because they trusted the person who recommended it to them. Efforts to recruit and engage participants without the assistance of partners were largely ineffective. For instance, promotores helped recruit almost all Spanish-speaking older adults, whereas other partners were instrumental in recruiting English-speaking older adults. This finding is consistent with prior work that highlighted that community-based partnerships can be crucial for populations who may face barriers to accessing care [50]. For example, a previous case study with older adults indicated that social workers could be suitable community partners because of their strong commitment to improve services for older adults, direct practical experience with barriers to care, and their skills for building collaborative relationships [51].

Promotores may have also played an important role in engaging Spanish-speaking participants throughout the program, as they have the cultural sensitivity and experience of the community. There is a need for mental health programs to be culturally appropriate [52], which ideally includes cultural vetting of recruitment, professional staff, and program materials. The fact that not all parts of myStrength were available in Spanish may have formed a barrier for Spanish-speaking participants in our study to fully engage with the platform. Nevertheless, an exploratory study in Marin County found that older adults still preferred myStrength over other mental health products, notably given the lack of suitable alternatives. This highlights the need to develop mental health programs that address the diverse cultural and linguistic backgrounds of the underserved communities.

Technology adoption is often influenced by the beliefs and recommendations of people close to the user, such as family, friends, or health providers [53]. This may be especially true for an older adult population who may have had little exposure to technology and may lack a mental model of what digital mental health entails. Digital mental health is a novel concept: it may be difficult to explain a digital mental health program to people who have little prior experience with technology and for them to envision how a mental health app may work. Therefore, participants may have been less likely to participate if it was advertised as a digital mental health program without endorsement from the people they trusted. Partnering with local organizations that have already established trust in communities can help reach specific populations.

Community partners can further play an important role in sustained engagement, as indicated by the finding that a large number of the participants discontinued myStrength after the program and support had ended. There may be opportunities in the future to make this type of support and community engagement an essential part of future staff job roles. In addition, prior work with a vulnerable youth population recommended integrating mental health services into services that youth are already receiving, such as schools [50]. There may be an opportunity to integrate aspects of a digital mental health program into existing home and community services that older adults are already receiving.

### Limitations

One limitation of our study pertains to missing data as not all participants completed each survey or survey question. Engaging older adults in research activities is a known challenge [54], and participants in our study at times could be overwhelmed by various program activities. Future programs should take this into consideration and keep survey instruments as brief as possible and offer different methods to complete surveys and interviews, for example, on the web, over the phone, or when possible, in person.

In addition, most of the participants were female. Men are often underrepresented in older adult health research programs, and future efforts may need to tailor recruitment strategies to increase male participation, for example, by featuring men in promotional material [55].

The digital mental health program studied in this paper was particularly focused on isolated (eg, geographically, culturally, and socially) older adults in specific communities with little access to mental health resources; therefore, caution must be exercised when generalizing the results. That said, we expect our findings to generalize to isolated older adults with low digital literacy.

The program offered staff support and myStrength to all participants and there was no comparison group. We did not know if the participants would have experienced similar benefits in the absence of this intervention.

Finally, this study was conducted during the COVID-19 pandemic, which may have exacerbated the level of isolation. It also added the challenge of providing support and digital literacy training remotely when participants were already struggling to get on the web in the first place. Future programs may offer in-person classes and provide more in-person support.

### Conclusions

DMHIs have the potential to improve mental health among older adults; however, factors such as low digital literacy and lack of support can form a barrier to starting and sustaining engagement. We found that ongoing support (eg, technical support, guided support, and social support) not only may be important from an implementation perspective to retain people in a digital health intervention but also may be more crucial in improving people’s experience and supporting their mental health than the technology itself. Having a social component and being able to participate in a program with other people may address loneliness and motivate people to enroll in a program that can help them stay connected with family and friends and improve their technical skills.

Future research should keep this in mind and provide a social support component, whenever possible. For example, web-based Zoom sessions can be held to connect with others using the platform, or a DMHI can provide the option to provide individual support and guidance from staff or coaches.

## Appendix

Multimedia Appendix 1 The survey instruments and interview protocol.

Multimedia Appendix 2 A screener form was used to assess participants’ eligibility to participate in the program.

## Abbreviations

DMHI: digital mental health intervention

## Data Availability

The survey instruments and interview protocol are included as supplementary materials in Multimedia Appendix 1. The data collected and analyzed during the study are not publicly available as they were not funding requirements, and the study participants did not consent for their data to be shared publicly.
