# Supplementary material for: Understanding the Role of Support in Digital Mental Health Programs With Older Adults: Users’ Perspective and Mixed Methods Study
Source: JMIR Form Res. 2022 Dec 13;6(12):e43192. doi: 10.2196/43192 (PMC9795392; doi:10.2196/43192)
Supplement: Multimedia Appendix 1 [file formative_v6i12e43192_app1.docx]

Understanding the role of support in digital mental health programs with older adults: the users’ perspective: Survey 1

Q71 Please review the study information sheet below. If you have any questions, you may contact *[enter contact information]*. Click on "Start" at the bottom of this page to begin the survey.

Q78 *[enter university contact information].*

You are being asked to participate in a research study. Participation in this study is voluntary. You may choose to skip a question or a study procedure. You may refuse to participate or discontinue your involvement at any time without penalty or loss of benefits.  You are free to withdraw from this study at any time.**If you decide to withdraw from this study, you should notify the research team immediately.**

You are being asked to participate in a research study to understand your experience with Marin County’s myStrength program. 

You are eligible to participate in this study if you are at least 18 years old and are enrolled in Marin County’s myStrength program.

We would like you to complete a survey to learn more about your experience using the myStrength application. The survey will last about 20 minutes.

Possible risks/discomforts associated with the study are uneasy feelings related to sharing information about your mental health and wellness.

There are no direct benefits from participation in the study.  However, this study may provide the following benefits to others or society: Improve the detection and acknowledgement of mental health symptoms sooner; Reduce stigma associated with mental illness by promoting mental wellness; Increase access to the appropriate level of support and care; Increase purpose, belonging, and social connectedness of individuals served; and, Improve mental health assessment tools and improvement in delivery of services.

There are no alternative procedures available.  The only alternative is not to participate in this study.

You will receive a $10 gift card to a local merchant for your participation in this study. There is no cost to you for participation in this study.

All research data collected will be stored securely and confidentially on a secure server. Information will be password protected and maintained in an encrypted format.

The research team, authorized UCI personnel, and regulatory entities, may have access to your study records to protect your safety and welfare.  While the research team will make every effort to keep your personal information confidential, it is possible that an unauthorized person might see it.  We cannot guarantee total privacy.

Future research use: Researchers will use your information to conduct this study. Once the study is done using your information, we may share them with other researchers so they can use them for other studies in the future. We will not share your name or any other private identifiable information that would let the researchers know who you are. We will not ask you for additional permission to share this de-identified information.

Data Retention: The researchers intend to keep the research data until the research is published and/or presented. If, during the course of this study, significant new information becomes available that may relate to your willingness to continue to participate, this information will be provided to you by the research team listed at the top of the form. If you have any comments, concerns, or questions regarding the conduct of this research please contact the researchers listed at the top of this form. It is important that you promptly tell the researchers if you believe that you have been injured because of taking part in this study.  You can tell the researcher in person or email him/her at the number listed at the top of this form.

Please contact the UCI Institutional Review Board by phone, (949) 824-6662, by e-mail at IRB@research.uci.eduor at 141 Innovation Drive, Suite 250, Irvine, CA 92697 if you are unable to reach the researchers listed at the top of the form and have general questions; have concerns or complaints about the research; have questions about your rights as a research subject; or have general comments or suggestions.

**What is an IRB?** An Institutional Review Board (IRB) is a committee made up of scientists and non-scientists. The IRB’s role is to protect the rights and welfare of human subjects involved in research.  The IRB also assures that the research complies with applicable regulations, laws, and institutional policies.

Q77 Click here to download a PDF version of the study information sheet.

End of Block: Study Information Sheet

Start of Block: Participant ID

Q2 Select your participant number:

▼ __________

End of Block: Participant ID

Start of Block: Program Participation

Q3 **The first part of the survey is going to ask you questions about your participation in the technology training program.**

Q4 Why did you decide to participate in this technology training program?

________________________________________________________________

________________________________________________________________

________________________________________________________________

________________________________________________________________

________________________________________________________________

Q5 How did you hear about this technology training program? (e.g., radio)

________________________________________________________________

________________________________________________________________

________________________________________________________________

________________________________________________________________

________________________________________________________________

End of Block: Program Particpation

Start of Block: General Technology Experience

Q6 **The next part of the survey is going to ask you questions about your experience using technology in general.**

Q7 Which of the following devices do you use? (Select all that apply.)

- Mac (7)
- PC (8)
- Mac (2)
- PC (9)
- iPhone (3)
- Android (10)
- iPad (11)
- Other (12)
- I don't use any of these. (6)
- Mobile phone but not a smartphone (5)

Display This Question:

If Q7 = iPhone

Or Q7 = Mobile phone but not a smartphone

Q8 The California LifeLine Program (California LifeLine) is a state program that provides discounted home phone and cell phone services to eligible households.


Do you participate in the California LifeLine program? (Select one answer.)

- Yes (1)
- No (2)
- I'm not sure. (3)
- I prefer not to answer. (4)

Q9 Do you have consistent access to WiFi at home? (Select one answer.)

- Yes (1)
- No (2)
- I'm not sure. (3)
- I prefer not to answer. (4)

Q10 Do you have a mobile data plan? (Select one answer.)

- Yes (1)
- No (2)
- I'm not sure. (3)
- I prefer not to answer. (4)

Q11 Where do you most often access internet? (Select one answer.)

- At home (1)
- At work (2)
- In public places (e.g., stores, library, etc.) (3)
- I never access the internet. (4)
- Other (please explain): (5) ________________________________________________
- I prefer not to answer. (6)

| Page Break |  |
| --- | --- |

Display This Question:

If Q11 != I never access the internet.

Q12 People may use the internet for streaming video/music, playing games, checking social media, using apps, browsing the web, etc. on a computer or phone or mobile device. 
 
**BEFORE the pandemic** (also known as COVID-19 or coronavirus) started: On a typical day, how often did you use the internet? (Select one answer.)

- Very frequently (1)
- Frequently (2)
- Occasionally (3)
- Rarely (4)
- Very rarely (5)
- I prefer not to answer. (6)

Display This Question:

If Q11 != I never access the internet.

Q13 People may use the internet for streaming video/music, playing games, checking social media, using apps, browsing the web, etc. on a computer or phone or mobile device. 
 
**SINCE the pandemic** (also known as COVID-19 or coronavirus) started: On a typical day, how often did you use the internet? (Select one answer.)

- Very frequently (1)
- Frequently (2)
- Occasionally (3)
- Rarely (4)
- Very rarely (5)
- I prefer not to answer. (6)

| Page Break |  |
| --- | --- |

Display This Question:

If Q11 != I never access the internet.

Q14 What are you comfortable using the internet for? (Select all that apply.)

- General information (1)
- Directions (2)
- Shopping (3)
- News (4)
- Email (5)
- Social media (6)
- Following blogs or interactive internet forums (7)
- Creating and maintaining my own website (8)
- Getting health information (9)
- Getting mental health information (10)
- Staying in contact with friends and/or family (11)
- Other (Please explain): (12) ________________________________________________

Q15 Which best describes your comfort level with protecting yourself on the internet? (Select one answer.)

- I have no idea how to protect myself while using the internet. (1)
- I know very little about protecting myself while using the internet. (2)
- I know something about protecting myself while using the internet, but I worry about accidentally giving away my privacy. (3)
- I am confident about protecting myself while using the internet. (4)

Q16 How do you use email? (Select all that apply.)

- I never use email. (1)
- I can send and receive emails. (2)
- I can attach a picture or document to an email. (3)
- I can download attachments and create email groups. (4)
- I can add new contacts. (5)

Q17 How do you keep track of your different passwords? (Select all that apply.)

- I do not have a set way. (1)
- I sometimes use post its or scraps of paper to write them down. (2)
- I use a single piece of paper or spreadsheet to write them on. (3)
- I use a password notebook. (4)
- I use an online password software. (5)
- Other (Please explain): (6) ________________________________________________

Q18 What is your level of understanding of using an app? (Select all that apply.)

- I don’t know what an app is. (1)
- I’ve never used an app. (2)
- I can use a few basic apps that came with my device, like email and Google. (3)
- I can use several different apps that came with my device. (4)
- I have downloaded a new app to my device. (5)
- I have downloaded several different apps, set up login credentials, and use them for a variety of purposes. (6)

Q19 Do you ever feel isolated or left out because you feel others can do things on a computer, smartphone, or tablet that you can’t? (Select one answer).

- Often (1)
- Sometimes (2)
- Never (3)

| Page Break |  |
| --- | --- |

Q20 Please rate the extent to which you agree or disagree, using a scale from **Strongly disagree (1)** to **Strongly agree (5)**.

|  | Strongly disagree-1 (1) | Somewhat disagree-2 (2) | Neither agree nor disagree-3 (3) | Somewhat agree-4 (4) | Strongly agree-5 (5) |
| --- | --- | --- | --- | --- | --- |
| I am confident using technology to look up information. (1) |  |  |  |  |  |
| I am confident using technology to support my well-being. (2) |  |  |  |  |  |
| I am interested in learning to use the internet. (3) |  |  |  |  |  |
| I am interested in learning to use a computer. (4) |  |  |  |  |  |
| I am interested in learning to use a smartphone or tablet. (5) |  |  |  |  |  |
| In general, I’m interested in using technology to support my mental health. (6) |  |  |  |  |  |
| I feel that as a result of my using technology, others know about me more than I am comfortable with. (7) |  |  |  |  |  |

| Page Break |  |
| --- | --- |

Q21 Please rate the extent to which you agree or disagree, using a scale from **Not confident at all (1)**to **5-Very confident (5).**

|  | Not at all confident-1 (1) | 2 (2) | Neutral-3 (3) | 4 (4) | Very confident-5 (5) |
| --- | --- | --- | --- | --- | --- |
| Removing a virus that infected your computer (1) |  |  |  |  |  |
| Learning how to use a new technology (2) |  |  |  |  |  |
| Using the Internet to gather information (3) |  |  |  |  |  |
| Participating in a discussion online (4) |  |  |  |  |  |
| Making new friends on the internet (5) |  |  |  |  |  |
| Uploading photos to a website (6) |  |  |  |  |  |
| Downloading and saving music (MP3) (7) |  |  |  |  |  |
| Judging the reliability of an online source (8) |  |  |  |  |  |
| Download apps to a mobile device (9) |  |  |  |  |  |
| Avoiding online scams (10) |  |  |  |  |  |

End of Block: General Technology Experience

Start of Block: Computer Experience

Q22 **The next part of the survey is going to ask you questions about your experience using a computer.**

Q23 Which best describes your comfort level with a computer? (Select one answer.)

- I am afraid to use it, and so I don’t use it at all. (1)
- l am nervous about using it, but I do use it once in a while. (2)
- I feel okay about it, use it sometimes but feel like I have a lot more I can learn. (3)
- I feel good about using my computer and use it often. (4)
- I am excited about using my computer and use it daily. (5)

Q24 Which of the following do you know how to do on the computer? (Select all that apply.)

- Turn it off and on (1)
- Logon with an account (2)
- Use the mouse (3)
- Connect to WiFi (4)
- Back up my files (5)
- Save and find files (6)
- Organize my files into folders and subfolders (7)
- Upload photos (8)
- Download software (9)
- Check and alter my settings (10)
- Word processing (11)
- System upgrades (12)
- Use email (13)
- Use the Internet (14)

Q25 What would you like to learn and be able to do with the computer? (Select all that apply.)

- Connect with friends and family (1)
- Connect with community (2)
- Get news, articles, or books (3)
- Learn new things and/or take online classes (4)
- Research topics I am interested in and/or share my interests with others who have the same interests (5)
- Use it for entertainment (e.g., watch movies or TV shows, listen to music, watch videos, play interactive games) (6)
- Get help with concerns such as depression, loneliness, anxiety or boredom (7)
- Get help with general health concerns (8)
- Other (Please explain): (9) ________________________________________________

End of Block: Computer Experience

Start of Block: Smartphone / Tablet Experience

Q26 **The next part of the survey is going to ask you questions about your experience using a smartphone or tablet.**

Q27 Which best describes your comfort level with a smartphone or tablet? (Select one answer.)

- I am afraid to use it, and so I don’t use it at all. (1)
- I am nervous about using it, but I do use it once in a while. (2)
- I feel okay about it, use it sometimes but feel like I have a lot more I can learn. (3)
- I feel good about using my smartphone and use it often. (4)
- I am excited about using my smartphone and use it daily. (5)

Q28 Which of the following do you know how to do on a smartphone or tablet? (Select all that apply.)

- Turn off and on (1)
- Logon to the Homescreen (2)
- Use the Home button (3)
- Connect to WiFi (4)
- Back up my device (5)
- Save and find photos (6)
- Enter Apple ID & Password (iPhone) or Google ID & Password (Android) (7)
- Download apps (8)
- Check and alter my settings (9)
- Use email (10)
- Use the internet (11)

Q29 What would you like to learn and be able to do with on a smartphone or tablet? (Select all that apply.)

- Connect with friends and family (1)
- Connect with community (2)
- Get news, articles, or books (3)
- Learn new things and/or take online classes (4)
- Research topics I am interested in and/or share my interests with others who have the same interests (5)
- Use it for entertainment (e.g., watch movies or TV shows, listen to music, watch videos, play interactive games) (6)
- Get help with concerns such as depression, loneliness, anxiety or boredom (7)
- Get help with general health concerns (8)
- Other (Please explain): (9) ________________________________________________

End of Block: Smartphone / Tablet Experience

Start of Block: Technology for Mental Health and Well-Being

Q30 **The next part of the survey is going to ask you questions about your opinion and experience using technology to support your mental health and well-being.**

Q31 In the past 12 months, have you tried to get help from an online tool, including mobile apps or texting services for problems with your mental health, emotions, nerves? (Select one.)

- Yes (1)
- No (2)
- I prefer not to answer. (3)

| Page Break |  |
| --- | --- |

Display This Question:

If Q31 = Yes

Q32 How useful was this online tool? (Select one.)

- Very (1)
- Somewhat (2)
- Not at all (3)
- I prefer not to answer. (4)

Display This Question:

If Q31 = No

Q33 What was the main reason you didn't try to get help from an online tool, including mobile apps or texting services? (Select one.)

- Got better / no longer needed (1)
- Wanted to handle problem myself (2)
- Don't own a smartphone or computer (3)
- Didn't know about these apps (4)
- Don't trust mobile apps (5)
- Concerns about privacy and security of data (6)
- Don't think it would be helpful or work (7)
- Cost (8)
- Don't have time (9)
- Received traditional / face-to-face services (10)
- Don't think I needed it (11)
- Don't have enough space to download new apps (12)
- Other (Please explain): (13) ________________________________________________
- I prefer not to answer. (14)

Q34 In the past 12 months, have you connected online with people that have mental health or alcohol/drug concerns similar to yours through methods such as social media, blogs, and online forums? (Select one answer.)

- Yes (1)
- No (2)
- I prefer not to answer (3)

Q35 In the past 12 months, have you used online tools to find, be referred to, contact, or connect with a mental health professional? (Select one answer.)

- Yes (1)
- No (2)
- I prefer not to answer (3)

Q36 When thinking about using technology to support your mental health, what aspects are important to you? (Select all that apply.)

- Availability in languages other than English (1)
- The app is free (2)
- The app is sensitive to my culture (3)
- My personal information will be kept private (4)
- The app will not have a negative effect on my device (examples: using the app will not drain my phone battery, using the app will not take up too much memory) (5)
- Parts of the app can be used offline (6)
- The app can be easily used by people with visual impairments (7)
- The app can be easily used by people who are deaf or hard of hearing (8)
- The app can be easily used by people with motor or coordination impairments (9)
- Other (Please explain): (10) ________________________________________________
- I prefer not to answer. (11)

End of Block: Technology for Mental Health and Well-Being

Start of Block: Connectedness, Loneliness, and Isolation

Q37 **The next set of questions ask about your feelings of connectedness, loneliness, and isolation.**   People may feel lonely or isolated even if they have people in their lives. For many, the pandemic has worsened feelings of loneliness because of the changes to our everyday lives. Some people may not be able to participate in typical in-person activities.

Q38 The next questions are about how you feel about different aspects of your life. For each one, please answer how often you feel that way.

Q39 First, how often do you feel that you lack companionship? Is it…

- Hardly ever (1)
- Some of the time (2)
- Often (3)

Q40 How often do you feel left out? Is it...

- Hardly ever (1)
- Some of the time (2)
- Often (3)

Q41 How often do you feel isolated from others? Is it...

- Hardly ever (1)
- Some of the time (2)
- Often (3)

Q42 Please select the number that best describes how many persons in your local area you feel you can depend on or feel close to.

How many persons in your local area do you feel like you can depend on or feel close to?

- None (1)
- 1 to 2 (2)
- More than 2 people (3)

Q43 The next questions are about interactions with others. For each question, please rate how much (or how often) you have participated in these activities during the past week. Unless specified otherwise, the activity can be in-person, by telephone, or online.

|  | None (1) | 1 to 2 times per week (2) | 3 to 6 times per week (3) | Daily (4) |
| --- | --- | --- | --- | --- |
| In the past week, about how often did you go to meetings of clubs, religious meetings, or other groups that you belong to? (in-person, by telephone, or online) (1) |  |  |  |  |
| How many times in the past week did you spend time **IN PERSON** with someone who does not live with you? (2) |  |  |  |  |
| How many times in the past week did you talk with friends or relatives on the **TELEPHONE**? (3) |  |  |  |  |
| How many times in the past week did you talk with friends or relatives using **VIDEO DEVICES** (such as FaceTime, Skype)? (4) |  |  |  |  |
| How many times in the past week did you communicate with friends or relatives using the **INTERNET** (such as e-mail, social media)? (5) |  |  |  |  |

Q44 What else would you like to share with us?


This includes but is not limited to sharing more information about your technology use, your mental health or wellness, or coping strategies and resources? If you prefer not to answer, then you can leave this blank.

________________________________________________________________

________________________________________________________________

________________________________________________________________

________________________________________________________________

________________________________________________________________

End of Block: Connectedness, Loneliness, and Isolation

Start of Block: Demographics

Q45 **The last part of the survey is going to ask about your background and demographics.** We ask this information to ensure we represent the viewpoints of your community.

Q46 What best describes your race/ethnicity? (Please select all that apply.)

- African American / Black (22)
- African (23)
- Jamaican (24)
- Haitian (25)
- Other African / Black (Please specify): (26) ________________________________________________
- American Indian (Please specify): (28) ________________________________________________
- Alaskan Native (Please specify): (29) ________________________________________________
- Asian Indian/South Asian (31)
- Cambodian (32)
- Chinese (33)
- Filipino (34)
- Japanese (35)
- Korean (36)
- Vietnamese (37)
- Other Asian (Please specify): (38) ________________________________________________
- Caribbean (40)
- Central American (41)
- Mexican/Mexican-American (42)
- Puerto Rican (43)
- Other Hispanic / Latino/a/x: (45) ________________________________________________
- South American (44)
- Iranian (47)
- Egyptian (48)
- Moroccan (49)
- Other Middle Eastern or North African (Please specify): (50) ________________________________________________
- Native Hawaiian (52)
- Other Pacific Islander (Please specify): (53) ________________________________________________
- Eastern European (55)
- European (56)
- Middle Eastern (57)
- Other White / Caucasian (Please specify): (58) ________________________________________________
- I prefer not to answer. (61)

| Page Break |  |
| --- | --- |

| 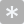 |
| --- |

Q47 How old are you (in years)?

________________________________________________________________

Q48 Are you a veteran? (Select one answer.)

- Yes (1)
- No (2)

| Page Break |  |
| --- | --- |

Q49 What language(s) do you speak fluently? (Select all that apply.)

- Arabic (1)
- Armenian (2)
- Cambodian (3)
- Cantonese (4)
- English (5)
- Farsi (6)
- Hmong (7)
- Korean (8)
- Mandarin (9)
- Other Chinese (10)
- Russian (11)
- Spanish (12)
- Tagalog (13)
- Vietnamese (14)
- American Sign Language (15)
- Other (Please explain): (16) ________________________________________________
- I prefer not to answer. (17)

Q50 What language do you prefer to use? (Select one answer.)

- Arabic (1)
- Armenian (2)
- Cambodian (3)
- Cantonese (4)
- English (5)
- Farsi (6)
- Hmong (7)
- Korean (8)
- Mandarin (9)
- Other Chinese (10)
- Russian (11)
- Spanish (12)
- Tagalog (13)
- Vietnamese (14)
- American Sign Language (15)
- Other (Please explain): (16) ________________________________________________
- I prefer not to answer. (17)

Q51 What is your gender? (Select one answer.)

- Man / male (1)
- Woman / female (2)
- Non-binary / Genderqueer / Gender non-conforming (3)
- Questioning or unsure of gender identity (4)
- I prefer to self-identify. (Please explain): (5) ________________________________________________
- I prefer not to answer. (6)

Q52 What sex were you assigned at birth?

- Male (1)
- Female (2)
- Other (Please explain): (3) ________________________________________________
- I prefer not to answer. (4)

Q53 Are you transgender?

- Yes (1)
- No (2)
- Questioning / unsure (3)
- I prefer not to answer. (4)

Q54 What is your sexual orientation? (Select one answer.)

- Gay or Lesbian (1)
- Heterosexual or Straight (2)
- Bisexual (3)
- Pansexual (4)
- Queer (5)
- Asexual (6)
- Questioning or unsure of sexual orientation (7)
- I prefer to self-identify (Please explain): (8) ________________________________________________
- I prefer not to answer. (9)

Q55
Do you have a disability? (Select one answer.) 


For this survey, disability is defined as a mental or physical impairment lasting more than 6 months and limiting major life activity but is not the result of a severe mental illness. 

- Yes (1)
- No (2)
- I prefer not to answer. (3)

| 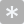 |
| --- |

Q56 What is your zip code?

________________________________________________________________

Q57 Are you currently experiencing homelessness? (Select one answer.)

- Yes (1)
- No (2)
- I prefer not to answer. (3)

Q58 What is your current yearly household income? (Select one answer.)

- Under $10,000 (1)
- $10,000-$19,999 (2)
- $20,000-$29,999 (3)
- $30,000-$39,999 (4)
- $40,000-$49,999 (5)
- $50,000-$59,999 (6)
- $60,000-$69,999 (7)
- $70,000-$79,999 (8)
- $80,000-$89,999 (9)
- $90,000-$99,999 (10)
- $100,000-$149,999 (11)
- $150,000 or above (12)

Q59 Please select the option that best describes your employment status. (Select one answer.)

- Employed full time (1)
- Employed part time (2)
- Unemployed looking for work (3)
- Unemployed not looking for work (4)
- Retired (5)
- Disabled (6)
- Other (Please explain): (7) ________________________________________________
- I prefer not to answer. (8)

Q60 What is the highest level of education you have completed? (Select one answer.)

- Less than high school (no diploma or GED) (1)
- High school graduate (or GED) (2)
- Some college with no degree (3)
- Associate’s degree (4)
- Bachelor’s degree (5)
- Graduate or professional degree (6)
- Other (Please explain): (7) ________________________________________________
- I prefer not to answer. (8)

| Page Break |  |
| --- | --- |

Q61 Please select the option that best describes your current marital status. (Select one answer.)

- Single (1)
- In a committed relationship or partnership but unmarried (2)
- Married (3)
- Widowed (4)
- Divorced (5)
- Separated (6)
- Other (Please explain): (7) ________________________________________________
- I prefer not to answer. (8)

Q62 What is your current living situation? (Select one answer.)

- I live alone. (1)
- I live with a spouse or partner. (2)
- I live with roommate(s). (3)
- I live with my children. (4)
- I live with family. (5)
- I live in an assisted living home. (6)
- Other (Please explain): (7) ________________________________________________
- I prefer not to answer (8)

Q63 Have you or anyone you know been diagnosed with the coronavirus/COVID-19? (Select one answer.).

- Yes (1)
- No (2)
- I prefer not to answer. (3)

Display This Question:

If Q63 = Yes

Q64 Who has been diagnosed? (Select all that apply.)

- Self (1)
- Partner/spouse (2)
- Child (3)
- Parent (4)
- Grandparent (5)
- Friend (6)
- Colleague (7)
- Acquaintance (8)
- Other (Please explain): (9) ________________________________________________
- I prefer not to answer. (10)

| Page Break |  |
| --- | --- |

Q65 Have you had a change in your employment because of the coronavirus/COVID-19? (Select one answer.)

- Lost my job (1)
- Had my hours reduced (2)
- Same job and hours, but working less due to children at home/other caregiving responsibilities (3)
- Working from home/remotely - my choice (4)
- Working from home/remotely - required by employment/government mandate (5)
- Other (Please explain): (6) ________________________________________________
- No change in my employment (7)
- I prefer not to answer. (8)

Q66 Please describe the economic impact of the coronavirus/COVID-19 on your family (Select all that apply.)

- Another member of the family has lost their job (1)
- Another member of the family has had their hours reduced (2)
- Another member of the family has the same job and hours, but working less due to children at home/other caregiving responsibilities (3)
- Another member of the family is working from home/remotely - required by employment/government mandate (4)
- We have gone from 2 earners to 1 (5)
- We have gone from 2 earners to 0 (6)
- We have gone from 1 earner to 0 (7)
- We have significantly reduced income (cannot quantify with choices listed above). (8)
- Other (Please explain): (9) ________________________________________________
- No economic impact (10)
- I prefer not to answer. (11)

End of Block: Demographics

Start of Block: Debrief

Q67 You have reached the end of the survey. Thank you so much for your participation! To submit your responses, select **"Submit responses"** at the bottom of this page. 

 Upon survey submission, you will be redirected to a form where you may enter your email address to receive a $10 eGift card for your participation. Your email address will NOT be linked to the information you provided in this survey.

 All information shared will be kept confidential. Findings from the survey will be used to help understand how the technology training program can best serve your community. 

 If you have any questions or concerns about the study, please contact *[enter contact information].*
   
 Please know resources are available to you if you choose to use them.

 **The Suicide Prevention Lifeline**
 They connect callers to trained counselors 24/7, for free. They also provide a chat function on their website. 
 Phone: 1-800-273-8255
 Website: https://suicidepreventionlifeline.org/

 **Crisis Text Line**
 It is free, 24/7 support for those who are feeling low.
 Text 741741 from anywhere in the US to text with a trained Crisis Counselor. 
 Website: https://www.crisistextline.org/

 **Marin County Behavioral Health and Recovery Services Access Line**
 The BHRS Access Line is open 24/7 to answer questions about services, arrange appointments, and make referrals.
 Phone: 1-888-818-1115

End of Block: Debrief

Survey 2

Start of Block: Study Information Sheet

Q1 Please review the study information sheet below. If you have any questions, you may contact *[enter contact information].* Click on "Start" at the bottom of this page to begin the survey.

*[enter university contact information].*

Please read the information below and ask questions about anything that you do not understand.  A researcher listed above will be available to answer your questions.

You are being asked to participate in a research study. Participation in this study is voluntary. You may choose to skip a question or a study procedure. You may refuse to participate or discontinue your involvement at any time without penalty or loss of benefits.  You are free to withdraw from this study at any time. If you decide to withdraw from this study, you should notify the research team immediately.

You are being asked to participate in a research study to understand your experience with Marin County’s myStrength program.   ·       

You are eligible to participate in this study if you are at least 18 years old and are enrolled in Marin County’s myStrength program.       

We would like you to complete a survey to learn more about your experience using the myStrength application. The survey will last about 20 minutes.

Possible risks/discomforts associated with the study are uneasy feelings related to sharing information about your mental health and wellness.  

There are no direct benefits from participation in the study.  However, this study may provide the following benefits to others or society: (1)Improve the detection and acknowledgement of mental health symptoms sooner; (2) Reduce stigma associated with mental illness by promoting mental wellness; (3) Increase access to the appropriate level of support and care; (4) Increase purpose, belonging, and social connectedness of individuals served; and,  (5) Improve mental health assessment tools and improvement in delivery of services.   ·       

There are no alternative procedures available.  The only alternative is not to participate in this study.     

You will receive a $10 gift card to a local merchant for your participation in this study.   ·      

There is no cost to you for participation in this study.    ·

All research data collected will be stored securely and confidentially on a secure server. Information will be password protected and maintained in an encrypted format.   ·

The research team, authorized UCI personnel, and regulatory entities, may have access to your study records to protect your safety and welfare.     While the research team will make every effort to keep your personal information confidential, it is possible that an unauthorized person might see it.  We cannot guarantee total privacy.

**Future research use**

Researchers will use your information to conduct this study. Once the study is done using your information, we may share them with other researchers so they can use them for other studies in the future. We will not share your name or any other private identifiable information that would let the researchers know who you are. We will not ask you for additional permission to share this de-identified information.

**Data Retention**

The researchers intend to keep the research data until the research is published and/or presented.

If, during the course of this study, significant new information becomes available that may relate to your willingness to continue to participate, this information will be provided to you by the research team listed at the top of the form.   ·

If you have any comments, concerns, or questions regarding the conduct of this research please contact the researchers listed at the top of this form.   ·

It is important that you promptly tell the researchers if you believe that you have been injured because of taking part in this study.  You can tell the researcher in person or email him/her at the number listed at the top of this form.   ·

Please contact the UCI Institutional Review Board by phone, (949) 824-6662, by e-mail at IRB@research.uci.eduor at 141 Innovation Drive, Suite 250, Irvine, CA 92697 if you are unable to reach the researchers listed at the top of the form and have general questions; have concerns or complaints about the research; have questions about your rights as a research subject; or have general comments or suggestions.

What is an IRB?  An Institutional Review Board (IRB) is a committee made up of scientists and non-scientists. The IRB’s role is to protect the rights and welfare of human subjects involved in research.  The IRB also assures that the research complies with applicable regulations, laws, and institutional policies.

Study_info_download Click to write the question text

End of Block: Study Information Sheet

Start of Block: Participant ID

Display This Question:

If If False

ID Please enter your Participant ID. If you do not know your Participant ID, skip to the next question.

________________________________________________________________

End of Block: Participant ID

Start of Block: Program Particpation

Q3 **The first part of the survey is going to ask you questions about your participation in the technology training program.**

Q4 Please select all technology training classes you participated in.

- **Class 1** Computer Literacy: I attended the class **live.** (1)
- **Class 1** Computer Literacy: I watched a **recording** of the class. (2)
- **Class 2** Computer Basics (part 1): I attended the class **live.** (3)
- **Class 2** Computer Basics (part 1): I watched a **recording** of the class. (4)
- **Class 3** Computer Basics (part 2): I attended the class **live.** (5)
- **Class 3** Computer Basics (part 2): I watched a **recording** of the class. (6)
- **Class 4** myStrength: I attended the class **live.** (7)
- **Class 4** myStrength: I watched a **recording** of the class. (8)
- I didn't participate in any live or recorded technology training class. (9)

Display This Question:

If Please select all technology training classes you participated in. != I didn't participate in any live or recorded technology training class.

Q5 Rate your level of satisfaction with the technology training you received. (Select one answer.)

- Very satisfied (1)
- Satisfied (2)
- Neither satisfied nor dissatisfied (3)
- Unsatisfied (4)
- Very unsatisfied (5)

| Page Break |  |
| --- | --- |

Display This Question:

If Please select all technology training classes you participated in. != I didn't participate in any live or recorded technology training class.

Q6 You said you were ${Q5/ChoiceGroup/SelectedChoices} with the technology training. Why did you choose this rating?

________________________________________________________________

________________________________________________________________

________________________________________________________________

________________________________________________________________

________________________________________________________________

| Page Break |  |
| --- | --- |

Q7 Please rate the extent to which you agree or disagree, using a scale from **Strongly disagree (1)** to **Strongly agree (5)**.

|  | Strongly disagree-1 (1) | Somewhat disagree-2 (2) | Neither agree nor disagree-3 (3) | Somewhat agree-4 (4) | Strongly agree-5 (5) | Does not apply to me (6) |
| --- | --- | --- | --- | --- | --- | --- |
| The technology training made me feel connected to other people. (1) |  |  |  |  |  |  |
| Because of the technology training, I am more likely to use technology. (2) |  |  |  |  |  |  |
| Because of the technology training, I am more likely to use technology to support my well-being. (3) |  |  |  |  |  |  |

End of Block: Program Particpation

Start of Block: General Technology Experience

Q8 **The next part of the survey is going to ask you questions about your experience using technology in general.**

Q9 Which of the following devices do you use? (Select all that apply.)

- Mac (1)
- PC (2)
- Mac (3)
- PC (4)
- iPhone (5)
- Android (6)
- iPad (7)
- Other (8)
- Mobile phone or cell phone but not a smartphone (9)
- I don't use any of these. (10)

Q10 Do you have consistent access to WiFi at home? (Select one answer.)

- Yes (1)
- No (2)
- I'm not sure. (3)
- I prefer not to answer. (4)

Q11 Do you have a mobile data plan? (Select one answer.)


A mobile data plan allows you to access the internet without WiFi.

- Yes (1)
- No (2)
- I'm not sure. (3)
- I prefer not to answer. (4)

Q12 Where do you most often access internet? (Select one answer.)

- At home (1)
- At work (2)
- In public places (e.g., stores, library, etc.) (3)
- I never access the internet. (4)
- Other (please explain:) (5) ________________________________________________
- I prefer not to answer. (6)

| Page Break |  |
| --- | --- |

Display This Question:

If Where do you most often access internet? (Select one answer.) != I never access the internet.

Q13 **Since participating in the technology training**, how often do you use the internet? (Select one answer).

- Very frequently (1)
- Frequently (2)
- Occasionally (3)
- Rarely (4)
- Very rarely (5)
- I have not used the internet since participating in the training (7)
- I prefer not to answer. (6)

| Page Break |  |
| --- | --- |

Display This Question:

If Where do you most often access internet? (Select one answer.) != I never access the internet.

Q14 What are you comfortable using the internet for? (Select all that apply.)

- General information (1)
- Directions (2)
- Shopping (3)
- News (4)
- Email (5)
- Social media (6)
- Following blogs or interactive internet forums (7)
- Creating and maintaining my own website (8)
- Getting health information (9)
- Getting mental health information (10)
- Staying in contact with friends and/or family (11)
- Other (Please explain): (12) ________________________________________________

Q15 Which best describes your comfort level with protecting yourself on the internet? (Select one answer.)

- I have no idea how to protect myself while using the internet. (1)
- I know very little about protecting myself while using the internet. (2)
- I know something about protecting myself while using the internet, but I worry about accidentally giving away my privacy. (3)
- I am confident about protecting myself while using the internet. (4)

Q16 How do you use email? (Select all that apply.)

- I never use email. (1)
- I can send and receive emails. (2)
- I can attach a picture or document to an email. (3)
- I can download attachments. (4)
- I can add new contacts. (5)
- I can create email groups. (6)

| Page Break |  |
| --- | --- |

Q17 How do you keep track of your different passwords? (Select all that apply.)

- I do not have a set way. (1)
- I sometimes use post its or scraps of paper to write them down. (2)
- I use a single piece of paper or spreadsheet to write them on. (3)
- I use a password notebook. (4)
- I use an online password software. (5)
- Other (Please explain): (6) ________________________________________________

Q18 What is your level of understanding of using an app? (Select all that apply.)

- I don’t know what an app is. (1)
- I’ve never used an app. (2)
- I can use a few basic apps that came with my device, like email and Google. (3)
- I can use several different apps that came with my device. (4)
- I have downloaded a new app to my device. (5)
- Someone has helped me download a new app to my device (7)
- Someone has helped me login credentials for a new app. (8)
- I have set up login credentials for a new app on my own. (9)
- I use apps for a variety of purposes. (10)

Q19 Do you ever feel isolated or left out because you feel others can do things on a computer, smartphone, or tablet that you can’t? (Select one answer).

- Often (1)
- Sometimes (2)
- Never (3)

| Page Break |  |
| --- | --- |

Q20 Please rate the extent to which you agree or disagree, using a scale from **Strongly disagree (1)** to **Strongly agree (5)**.

|  | Strongly disagree-1 (1) | Somewhat disagree-2 (2) | Neither agree nor disagree-3 (3) | Somewhat agree-4 (4) | Strongly agree-5 (5) |
| --- | --- | --- | --- | --- | --- |
| I am confident using technology to look up information. (1) |  |  |  |  |  |
| I am confident using technology to support my well-being. (2) |  |  |  |  |  |
| I am interested in learning to use the internet. (3) |  |  |  |  |  |
| I am interested in learning to use a computer. (4) |  |  |  |  |  |
| I am interested in learning to use a smartphone or tablet. (5) |  |  |  |  |  |
| In general, I’m interested in using technology to support my mental health. (6) |  |  |  |  |  |
| I feel that as a result of my using technology, others know about me more than I am comfortable with. (7) |  |  |  |  |  |

| Page Break |  |
| --- | --- |

Q21 The next set of statements are going to ask you**how confident you feel in your ability** to do the following things. Please rate how confident you are, using a scale from **Not confident at all / Not able to do at all (1)**  to**5-Very confident (5) / Able to do on my own.**

|  | Not at all confident-1 (1) | 2 (2) | Neutral-3 (3) | 4 (4) | Very confident-5 (5) |
| --- | --- | --- | --- | --- | --- |
| Removing a virus that infected your computer (1) |  |  |  |  |  |
| Learning how to use a new technology (2) |  |  |  |  |  |
| Using the Internet to gather information (3) |  |  |  |  |  |
| Participating in a discussion online (4) |  |  |  |  |  |
| Making new friends on the internet (5) |  |  |  |  |  |
| Uploading photos to a website (6) |  |  |  |  |  |
| Downloading and saving music (MP3) (7) |  |  |  |  |  |
| Judging the reliability of an online source (8) |  |  |  |  |  |
| Download apps to a mobile device (9) |  |  |  |  |  |
| Avoiding online scams (10) |  |  |  |  |  |

End of Block: General Technology Experience

Start of Block: Computer Experience

Q22 **The next part of the survey is going to ask you questions about your experience using a computer.**

Q23 How often do you use a computer (select one answer.)

- Never (1)
- Rarely (2)
- Sometimes (3)
- Often (4)
- Always (5)
- Other (please explain) (6) ________________________________________________

Q24 Which of the following do you know how to do on the computer? (Select all that apply.)

- Turn it off and on (1)
- Logon with an account (2)
- Use the mouse (3)
- Connect to WiFi (4)
- Back up my files (5)
- Save and find files (6)
- Organize my files into folders and subfolders (7)
- Upload photos (8)
- Download software (9)
- Check and alter my settings (10)
- Word processing (11)
- System upgrades (12)
- Use email (13)
- Use the Internet (14)

End of Block: Computer Experience

Start of Block: Smartphone / Tablet Experience

Q25 **The next part of the survey is going to ask you questions about your experience using a smartphone or tablet.**

Q26 How often do you use a smartphone or tablet? (Select one answer.)

- Never (1)
- Rarely (2)
- Sometimes (3)
- Often (4)
- Always (5)
- Other (please explain) (6) ________________________________________________

Q27 Which of the following do you know how to do on a smartphone or tablet? (Select all that apply.)

- Turn off and on (1)
- Logon to the Homescreen (2)
- Use the Home button (3)
- Connect to WiFi (4)
- Back up my device (5)
- Save and find photos (6)
- Enter Apple ID & Password (iPhone) or Google ID & Password (Android) (7)
- Download apps (8)
- Check and alter my settings (9)
- Use email (10)
- Use the internet (11)

End of Block: Smartphone / Tablet Experience

Start of Block: Technology for Mental Health and Well-Being

Q28  **The next part of the survey is going to ask you questions about your opinion and experience using technology to support your mental health and well-being.**

Q29 There are many ways someone may get support for their well-being or mental health online. 

 **In the past 4 weeks**, have you tried to get help from an online tool **other than myStrength**, including mobile apps or texting services for problems with your mental health, emotions, nerves? (Select one answer.)

- Yes (1)
- No (2)
- I prefer not to answer. (3)

| Page Break |  |
| --- | --- |

Display This Question:

If There are many ways someone may get support for their well-being or mental health online. In the... = Yes

Q30 How useful was this online tool? (Select one answer.)

- Very (1)
- Somewhat (2)
- Not at all (3)
- I prefer not to answer. (4)

Display This Question:

If There are many ways someone may get support for their well-being or mental health online. In the... = No

Q31 What was the main reason you didn't try to get help from an online tool, including mobile apps or texting services? (Select one answer.)

- Got better / no longer needed (1)
- Wanted to handle problem myself (2)
- Don't own a smartphone or computer (3)
- Didn't know about these apps (4)
- Don't trust mobile apps (5)
- Concerns about privacy and security of data (6)
- Don't think it would be helpful or work (7)
- Cost (8)
- Don't have time (9)
- Received traditional / face-to-face services (10)
- Don't think I needed it (11)
- Don't have enough space to download new apps (12)
- Other (Please explain): (13) ________________________________________________
- I prefer not to answer. (14)

Q32 In the past 4 weeks, have you connected online with people that have mental health or alcohol/drug concerns similar to yours through methods such as social media, blogs, and online forums? (Select one answer.)

- Yes (1)
- No (2)
- I prefer not to answer (3)

Q33 In the past 4 weeks, have you used online tools to find, be referred to, contact, or connect with a mental health professional? (Select one answer.)

- Yes (1)
- No (2)
- I prefer not to answer (3)

End of Block: Technology for Mental Health and Well-Being

Start of Block: Expectations of myStrength

Q34 **The next set of questions are going to ask you questions about your expectations of myStrength.**

Q35 Please rate the extent to which you agree or disagree, using a scale from **Strongly disagree (1)** to **Strongly agree (5)**.

|  | Strongly disagree-1 (1) | Somewhat disagree-2 (2) | Neither agree nor disagree-3 (3) | Somewhat agree-4 (4) | Strongly agree-5 (5) |
| --- | --- | --- | --- | --- | --- |
| I believe myStrength will be useful in my daily life. (1) |  |  |  |  |  |
| I believe using myStrength will meet my mental health needs. (2) |  |  |  |  |  |
| I believe using myStrength will improve my mental health. (3) |  |  |  |  |  |

End of Block: Expectations of myStrength

Start of Block: Well-Being and Health

Q36 **The next part of the survey is going to ask about your well-being and health.**

Q37 The next set of statements are going to ask you about how you have been feeling during the past 30 days. For each statement, please rate how often you have been feeling that way over the past 30 days, on a scale from **None of the time (1)** to **All of the time (5).**
   
During the last 30 days, about how often did you feel...

|  | None of the time-1 (1) | A little of the time-2 (2) | Some of the time-3 (3) | Most of the time-4 (4) | All of the time-5 (5) |
| --- | --- | --- | --- | --- | --- |
| ...tired out for no good reason? (1) |  |  |  |  |  |
| ...nervous? (2) |  |  |  |  |  |
| ...so nervous that nothing could calm you down? (3) |  |  |  |  |  |
| ...hopeless? (4) |  |  |  |  |  |
| ...restless or fidgety? (5) |  |  |  |  |  |
| ...so restless you could not sit still? (6) |  |  |  |  |  |
| ...depressed? (7) |  |  |  |  |  |
| ...that everything was an effort? (8) |  |  |  |  |  |
| ...so sad that nothing could cheer you up? (9) |  |  |  |  |  |
| ...worthless? (10) |  |  |  |  |  |

Q38
Many people have experienced (or are currently experiencing) a mental health challenge that impacts their ability to function in their family, relationships, or workplace.  Many different terms are used to refer to those experiences—some terms include “mental illness”, “mental health challenge”, “emotional distress,” “psychological disorder,” “mental health condition,” etc. 

Have you or do experience mental health challenges or concerns? (Select one.)

- Yes, I have been diagnosed with a mental health condition (1)
- Yes, I experience mental health challenges but have not been diagnosed by a professional (2)
- No, I don't experience mental health challenges (3)
- Other (Please explain): (4) ________________________________________________
- I prefer not to answer. (5)

Q39 The next set of questions are about mental health stigma. Stigma can cause people to feel badly for something that is out of their control. Keep in mind, these statements don’t represent our views of mental illness. 


Throughout this section, the term “mental illness” will be used. However, there are other terms that could be used, such as mental health challenge,  psychological disorder, or mental health condition.

Display This Question:

If Many people have experienced (or are currently experiencing) a mental health challenge that impac... = Yes, I have been diagnosed with a mental health condition

Or Many people have experienced (or are currently experiencing) a mental health challenge that impac... = Yes, I experience mental health challenges but have not been diagnosed by a professional

Q40 Please rate the extent to which you agree or disagree, using a scale from **Strongly disagree (1)** to **Strongly agree (5)**.

|  | Strongly disagree-1 (1) | Somewhat disagree-2 (2) | Neither agree nor disagree-3 (3) | Somewhat agree-4 (4) | Strongly agree-5 (5) |
| --- | --- | --- | --- | --- | --- |
| I feel out of place in the world because I have a mental illness (4) |  |  |  |  |  |
| Having a mental illness has spoiled my life (6) |  |  |  |  |  |
| People without a mental illness could not possibly understand me (7) |  |  |  |  |  |
| I am embarrassed or ashamed that I have a mental illness (16) |  |  |  |  |  |
| I am disappointed in myself for having a mental illness (15) |  |  |  |  |  |
| I feel inferior to others who don’t have a mental illness (17) |  |  |  |  |  |
| I don’t talk about myself much because I don’t want to burden others with my mental illness (18) |  |  |  |  |  |
| I don’t socialize as much as I used to because my mental illness might make me look or behave ‘weird’ (19) |  |  |  |  |  |
| Negative stereotypes about mental illness keep me isolated from the ‘normal’ world (20) |  |  |  |  |  |
| I stay away from social situations in order to protect my family or friends from embarrassment (21) |  |  |  |  |  |
| Being around people who don’t have a mental illness makes me feel out of place or inadequate (22) |  |  |  |  |  |
| I avoid getting close to people who don’t have a mental illness to avoid rejection (23) |  |  |  |  |  |
| Coping with my mental illness is no longer the main focus of my life (24) |  |  |  |  |  |
| My symptoms interfere less and less with my life (25) |  |  |  |  |  |
| My symptoms seem to be a problem for shorter periods of time each time they occur (26) |  |  |  |  |  |

Q41 Please rate the extent to which you agree or disagree, using a scale from **Strongly disagree (1)** to **Strongly agree (5)**.

|  | Strongly disagree-1 (1) | Somewhat disagree-2 (2) | Neither agree nor disagree-3 (3) | Somewhat agree-4 (4) | Strongly agree-5 (5) |
| --- | --- | --- | --- | --- | --- |
| I know when to ask for help (1) |  |  |  |  |  |
| I am willing to ask for help (2) |  |  |  |  |  |
| I ask for help when I need it (3) |  |  |  |  |  |

End of Block: Well-Being and Health

Start of Block: Connectedness, Loneliness, and Isolation

Q42 **The next set of questions ask about your feelings of connectedness, loneliness, and isolation.**   People may feel lonely or isolated even if they have people in their lives. For many, the pandemic has worsened feelings of loneliness because of the changes to our everyday lives. Some people may not be able to participate in typical in-person activities.

| Page Break |  |
| --- | --- |

Q43 The next questions are about how you feel about different aspects of your life. For each one, please answer how often you feel that way.

Q44 First, how often do you feel that you lack companionship? Is it…

- Hardly ever (1)
- Some of the time (2)
- Often (3)

Q45 How often do you feel left out? Is it...

- Hardly ever (1)
- Some of the time (2)
- Often (3)

Q46 How often do you feel isolated from others? Is it...

- Hardly ever (1)
- Some of the time (2)
- Often (3)

| Page Break |  |
| --- | --- |

Q47 The following statements describe how people sometimes feel. For each statement, please indicate how often you feel the way described by selecting a number in the space provided, on a scale from **Never (1)** to **Always (4)**. Here is an example:  

 How often do you feel happy?   If you never felt happy, you would respond "never"; if you always feel happy, you would respond "always!'    

 How often do you feel...

|  | Never-1 (1) | Rarely-2 (2) | Sometimes-3 (3) | Always-4 (4) |
| --- | --- | --- | --- | --- |
| ...that you are "in tune" with the people around you? (1) |  |  |  |  |
| ...that you lack companionship? (2) |  |  |  |  |
| ...that there is no one you can turn to? (3) |  |  |  |  |
| ...alone? (4) |  |  |  |  |
| ...part of a group of friends? (5) |  |  |  |  |
| ...that you have a lot in common with the people around you? (6) |  |  |  |  |
| ...that you are no longer close to anyone? (7) |  |  |  |  |
| ...that your interests and ideas are not shared by those around you? (8) |  |  |  |  |
| ...outgoing and friendly? (9) |  |  |  |  |
| ...close to people? (10) |  |  |  |  |
| ...left out? (11) |  |  |  |  |
| ...that your relationships with others are not meaningful? (12) |  |  |  |  |
| ...that no one really knows you well? (13) |  |  |  |  |
| ...isolated from others? (14) |  |  |  |  |
| ...you can find companionship when you want it? (15) |  |  |  |  |
| ...that there are people who really understand you? (16) |  |  |  |  |
| ...shy? (17) |  |  |  |  |
| ...that people are around you but not with you? (18) |  |  |  |  |
| ...that there are people you can talk to? (19) |  |  |  |  |
| ...that there are people you can turn to? (20) |  |  |  |  |

| Page Break |  |
| --- | --- |

Q48 Please select the number that best describes how many persons in your local area you feel you can depend on or feel close to.


How many persons in your local area do you feel like you can depend on or feel close to?

- None (1)
- 1 to 2 (2)
- More than 2 people (3)

Q49 The next questions are about interactions with others. For each question, please rate how much (or how often) you have participated in these activities during the past week. Unless specified otherwise, the activity can be in-person, by telephone, or online.

|  | None (1) | 1 to 2 times per week (2) | 3 to 6 times per week (3) | Daily (4) |
| --- | --- | --- | --- | --- |
| In the past week, about how often did you go to meetings of clubs, religious meetings, or other groups that you belong to? (in-person, by telephone, or online, such as using Zoom) (1) |  |  |  |  |
| How many times in the past week did you spend time **IN PERSON** with someone who does not live with you? (2) |  |  |  |  |
| How many times in the past week did you talk with friends or relatives on the **TELEPHONE?** (3) |  |  |  |  |
| How many times in the past week did you talk with friends or relatives using **VIDEO DEVICES** (such as FaceTime, Skype)? (4) |  |  |  |  |
| How many times in the past week did you communicate with friends or relatives using the **INTERNET** (such as e-mail, social media)? (5) |  |  |  |  |

| Page Break |  |
| --- | --- |

Q50 When do you need mental health support the most? (Select all that apply.)

- During the day (1)
- At night (2)
- At home (3)
- At school (4)
- At work (5)
- Before or during a social situation (6)
- When dealing with a significant life event (e.g., after a breakup, death or illness in my family) (7)
- Other (Please explain): (8) ________________________________________________
- I prefer not to answer. (9)

End of Block: Connectedness, Loneliness, and Isolation

Start of Block: Mental Healthcare Services

Q51 Do you currently have health insurance?

- Yes (1)
- No (2)
- I prefer not to answer. (3)

| Page Break |  |
| --- | --- |

Display This Question:

If Do you currently have health insurance?  = Yes

Q52 Does your health insurance plan provide any coverage for visits to a mental health professional (psychiatrist, psychologist, clinical social worker, etc.)? (Select one answer.)

- I have no idea. (1)
- I think it would not, but I am not too sure. (2)
- No, it definitely would not. (3)
- Yes, it definitely would. (4)
- I think it would, but I am not too sure. (5)

Q53 In the past 12 months, have you visited an Emergency Department (ED) for problems related to your mental health, emotions, or nerves? (Select one answer.)

- Yes (1)
- No (2)
- I don't know. (3)
- I prefer not to answer. (4)

Q54 In the past 12 months, have you been hospitalized for problems related to your mental health, emotions, or nerves? (Select one answer.)

- Yes (1)
- No (2)
- I don't know. (3)
- I prefer not to answer. (4)

Q55 In the past 12 months, how frequently have you used professional health services (psychiatrist, psychologist, clinical social worker, etc.) for problems related to your mental health, emotions, or nerves? This can refer to an in-person visit or remote contact such as a phone call or videocall. (Select one answer.)

- Daily (1)
- Weekly (2)
- Monthly (3)
- A few times a year (4)
- Never (5)
- I don’t know. (6)
- I prefer not to answer. (7)

Q56 Do you currently have medication prescribed to you by a professional (psychiatrist, psychologist, clinical social worker, etc.) for problems related to your mental health, emotions, or nerves? (Select one.)

- Yes (1)
- No (2)
- I prefer not to answer. (3)

End of Block: Mental Healthcare Services

Start of Block: Demographics

Q57 What is your current living situation? (Select one answer.)

- I live alone. (1)
- I live with a spouse or partner. (2)
- I live with roommate(s). (3)
- I live with my children. (4)
- I live with family. (5)
- I live in an assisted living home. (6)
- Other (Please explain): (7) ________________________________________________
- I prefer not to answer (8)

Q58 What else would you like to share with us?


This includes but is not limited to sharing more information about your thoughts on this program, your mental health or wellness, or coping strategies and resources. If you prefer not to answer, then you can leave this blank.

________________________________________________________________

________________________________________________________________

________________________________________________________________

________________________________________________________________

________________________________________________________________

End of Block: Demographics

Start of Block: Debrief

Q59
You have reached the end of the survey. Thank you so much for your participation! To submit your responses, select "**Submit responses**" at the bottom of this page. 

Upon survey submission, you will be redirected to a form where you may enter your email address to receive a $10 eGift card for your participation. Your email address will NOT be linked to the information you provided in this survey. All information shared will be kept confidential. Findings from the survey will be used to help understand how the technology training program can best serve your community. 

If you have any questions or concerns about the study, please contact *[enter contact information].*
   
 Please know resources are available to you if you choose to use them.

 **The Suicide Prevention Lifeline**
 They connect callers to trained counselors 24/7, for free. They also provide a chat function on their website. 
 Phone: 1-800-273-8255
 Website: <https://suicidepreventionlifeline.org/>

 **Crisis Text Line**
 It is free, 24/7 support for those who are feeling low.
 Text 741741 from anywhere in the US to text with a trained Crisis Counselor. 
 Website: <https://www.crisistextline.org/>

 **Marin County Behavioral Health and Recovery Services Access Line**
 The BHRS Access Line is open 24/7 to answer questions about services, arrange appointments, and make referrals.
 Phone: 1-888-818-1115

End of Block: Debrief

Survey 3

Start of Block: Study Information Sheet

Q1 Please review the study information sheet below. If you have any questions, you may contact *[enter contact information]*. Click on "Start" at the bottom of this page to begin the survey.

Q2
*[enter university contact information].*

·       Please read the information below and ask questions about anything that you do not understand.  A researcher listed above will be available to answer your questions.

 ·       You are being asked to participate in a research study. Participation in this study is voluntary. You may choose to skip a question or a study procedure. You may refuse to participate or discontinue your involvement at any time without penalty or loss of benefits.  You are free to withdraw from this study at any time. If you decide to withdraw from this study, you should notify the research team immediately.

 ·       You are being asked to participate in a research study to understand your experience with Marin County’s myStrength program.

 ·       You are eligible to participate in this study if you are at least 18 years old and are enrolled in Marin County’s myStrength program.

 ·       We would like you to complete a survey to learn more about your experience using the myStrength application. The survey will last about 20 minutes. 

 ·       Possible risks/discomforts associated with the study are uneasy feelings related to sharing information about your mental health and wellness.

 ·       There are no direct benefits from participation in the study.  However, this study may provide the following benefits to others or society: (1)Improve the detection and acknowledgement of mental health symptoms sooner; (2) Reduce stigma associated with mental illness by promoting mental wellness; (3) Increase access to the appropriate level of support and care; (4) Increase purpose, belonging, and social connectedness of individuals served; and,  (5) Improve mental health assessment tools and improvement in delivery of services.

 ·       There are no alternative procedures available.  The only alternative is not to participate in this study.

 ·       You will receive a $10 gift card to a local merchant for your participation in this study.

 ·       There is no cost to you for participation in this study. 

 ·       All research data collected will be stored securely and confidentially on a secure server. Information will be password protected and maintained in an encrypted format.

 ·       The research team, authorized UCI personnel, and regulatory entities, may have access to your study records to protect your safety and welfare.  

 While the research team will make every effort to keep your personal information confidential, it is possible that an unauthorized person might see it.  We cannot guarantee total privacy.
                    
 Future research use
 ·       Researchers will use your information to conduct this study. Once the study is done using your information, we may share them with other researchers so they can use them for other studies in the future. We will not share your name or any other private identifiable information that would let the researchers know who you are. We will not ask you for additional permission to share this de-identified information.

 Data Retention
 ·       The researchers intend to keep the research data until the research is published and/or presented

 ·       If, during the course of this study, significant new information becomes available that may relate to your willingness to continue to participate, this information will be provided to you by the research team listed at the top of the form.

 ·       If you have any comments, concerns, or questions regarding the conduct of this research please contact the researchers listed at the top of this form.

 ·       It is important that you promptly tell the researchers if you believe that you have been injured because of taking part in this study.  You can tell the researcher in person or email him/her at the number listed at the top of this form.

 ·       Please contact the UCI Institutional Review Board by phone, (949) 824-6662, by e-mail at [IRB@research.uci.edu](mailto:IRB@research.uci.edu) or at 141 Innovation Drive, Suite 250, Irvine, CA 92697 if you are unable to reach the researchers listed at the top of the form and have general questions; have concerns or complaints about the research; have questions about your rights as a research subject; or have general comments or suggestions.

 What is an IRB?  An Institutional Review Board (IRB) is a committee made up of scientists and non-scientists. The IRB’s role is to protect the rights and welfare of human subjects involved in research.  The IRB also assures that the research complies with applicable regulations, laws, and institutional policies. 

Q130 Click to write the question text

End of Block: Study Information Sheet

Start of Block: Participant ID

Display This Question:

If If False

Q4 Please enter your participant ID number. If you do not know your participant ID, please skip to the next question.

________________________________________________________________

End of Block: Participant ID

Start of Block: General Technology Experience

Q5 **The first part of the survey is going to ask you questions about your experience using technology in general.**

Q9 Please rate the extent to which you agree or disagree, using a scale from **Strongly disagree (1)** to **Strongly agree (5)**.

|  | Strongly disagree-1 (1) | Somewhat disagree-2 (2) | Neither agree nor disagree-3 (3) | Somewhat agree-4 (4) | Strongly agree-5 (5) |
| --- | --- | --- | --- | --- | --- |
| I am confident using technology to look up information. (1) |  |  |  |  |  |
| I am confident using technology to support my well-being. (2) |  |  |  |  |  |
| I feel that as a result of my using technology, others know about me more than I am comfortable with. (4) |  |  |  |  |  |

End of Block: General Technology Experience

Start of Block: Technology for Mental Health and Well-Being

Q10 **The next part of the survey is going to ask you questions about your opinion and experience using technology to support your mental health and well-being.**

Q11
There are many ways someone may get support for their well-being and mental health online. 
  **In the past 2 months**, have you tried to get help from an online tool **other than myStrength**, including mobile apps or texting services for problems with your mental health, emotions, nerves? (Select one answer.)

- Yes (1)
- No (2)
- I prefer not to answer. (3)

Display This Question:

If There are many ways someone may get support for their well-being and mental health online.    In... = Yes

Q12 How useful was this online tool? (Select one answer.)

- Very (1)
- Somewhat (2)
- Not at all (3)
- I prefer not to answer. (4)

| Page Break |  |
| --- | --- |

Display This Question:

If There are many ways someone may get support for their well-being and mental health online.    In... = No

Q13 What was the main reason you didn't try to get help from an online tool (other than myStrength), including mobile apps or texting services? (Select one answer.)

- Got better / no longer needed (1)
- Wanted to handle problem myself (2)
- Don't own a smartphone or computer (3)
- Didn't know about these apps (4)
- Don't trust mobile apps (5)
- Concerns about privacy and security of data (6)
- Don't think it would be helpful or work (7)
- Cost (8)
- Don't have time (9)
- Received traditional / face-to-face services (10)
- Don't think I needed it (11)
- Don't have enough space to download new apps (12)
- Other (Please explain): (13) ________________________________________________
- I prefer not to answer. (14)

| Page Break |  |
| --- | --- |

Q14 **In the past 2 months**, have you connected online with people that have mental health or alcohol/drug concerns similar to yours through methods such as social media, blogs, and online forums? (Select one answer.)

- Yes (1)
- No (2)
- I prefer not to answer (3)

Q15 **In the past 2 months**, have you used online tools to find, be referred to, contact, or connect with a mental health professional? (Select one answer.)

- Yes (1)
- No (2)
- I prefer not to answer (3)

End of Block: Technology for Mental Health and Well-Being

Start of Block: Program Combined Particpation

Q16 **The next part of the survey is going to ask you questions about your thoughts about the program overall. The program refers to the COMBINED technology training, options for access to a nurse intern, and access to myStrength.**

Q17 Please rate the extent to which you agree or disagree, using a scale from **Strongly disagree (1)** to **Strongly agree (5)**.

|  | Strongly disagree-1 (1) | Somewhat disagree-2 (2) | Neither agree nor disagree-3 (3) | Somewhat agree-4 (4) | Strongly agree-5 (5) | Not applicable (6) |
| --- | --- | --- | --- | --- | --- | --- |
| Participating in this program made me feel connected to other people. (1) |  |  |  |  |  |  |
| Because of this program, I am more likely to use technology to support my well-being. (5) |  |  |  |  |  |  |

| Page Break |  |
| --- | --- |

Q18 Please rate how much support you received to use myStrength from each of the following people:

|  | Too little support (15) | The right amount of support (16) | Too much support (17) | Not applicable (14) |
| --- | --- | --- | --- | --- |
| Staff from Marin County, for example Lorraine, Damaris, Chandrika (1) |  |  |  |  |
| Nurse intern(s) (2) |  |  |  |  |
| Promotores (3) |  |  |  |  |
| Friends or family (4) |  |  |  |  |

End of Block: Program Combined Particpation

Start of Block: myStrength Experience

Q19 **The next part of the survey is going to ask you questions about your thoughts about myStrength.**

Q20 Approximately how long have you used myStrength? (Select one answer.)

- Less than one week (1)
- A couple of weeks (2)
- About a month (3)
- About 2 months (4)
- More than 2 months (5)

Q21 What best describes your use of myStrength? (Select one answer.)

- Daily (7)
- Several times a week (8)
- Rarely (9)
- I only used it once (10)
- I never used it (11)
- Other (Please explain): (12) ________________________________________________

Display This Question:

If What best describes your use of myStrength? (Select one answer.) = I never used it

Q22 Why did you not use myStrength? (Select all that apply.)

- I thought myStrength would be too difficult to use. (1)
- I thought myStrength would take up too much of my time. (2)
- I thought myStrength would cost too much. (3)
- I thought myStrength wouldn't be useful. (4)
- I wouldn’t have been able to get help from others if I had difficulties using myStrength. (5)
- I was worried what other people might think of me if I used myStrength. (6)
- I was concerned that my data would not be private on myStrength. (7)
- I was concerned myStrength wouldn’t understand my culture. (8)
- myStrength was not in a language that I want to use. (9)
- I didn't have consistent access to a smartphone or internet connection to use myStrength. (10)
- I didn't have enough space on my phone or tablet to use myStrength. (11)
- I wanted to handle the problem myself. (12)
- I felt like I didn't need myStrength. (13)
- I was using other technologies to support my mental health that work well for me. (14)
- I was using other strategies to support my mental health that work well for me. (15)
- I only wanted to use traditional mental health services. (16)
- Other (Please explain): (17) ________________________________________________

End of Block: myStrength Experience

Start of Block: myStrength Experience for Users

Display This Question:

If What best describes your use of myStrength? (Select one answer.) != I never used it

Q23 Which of the following devices do you or did you use to access myStrength? (Select all that apply.)

- Desktop (1)
- Laptop computer (2)
- Smartphone (3)
- Tablet (e.g., iPad) (4)
- Mobile phone but not a smartphone (5)
- None of the above (6)

| Page Break |  |
| --- | --- |

Display This Question:

If Which of the following devices do you or did you use to access myStrength? (Select all that apply.) = Tablet (e.g., iPad)

Q24 Is the tablet you're using given to you by Marin County?

- Yes (1)
- No (2)
- I'm not sure. (3)
- I prefer not to answer. (4)

Display This Question:

If What best describes your use of myStrength? (Select one answer.) != I never used it

Q25 What language did you use myStrength in? (Select one answer.)

- Spanish (1)
- English (2)
- Both Spanish and English (3)

Display This Question:

If What best describes your use of myStrength? (Select one answer.) != I never used it

Q26 During the period that you were using myStrength **for ${Q20/ChoiceGroup/SelectedChoices}**, have you stopped using myStrength? (Select one answer.)

- Yes (1)
- No (2) Display This Question:

If During the period that you were using myStrength for ${q://QID127/ChoiceGroup/SelectedChoices}, h... = Yes

Q27 Why did you stop using or take a break from myStrength? (Select all that apply.)

- myStrength was too difficult to use. (1)
- I lost access to myStrength and couldn't figure out how to get it back. (2)
- myStrength took up too much of my time. (4)
- myStrength costs too much. (5)
- myStrength was not useful. (6)
- I was worried what other people might think of me if they knew I used myStrength. (7)
- I was concerned that my data was not private on myStrength. (8)
- myStrength didn’t understand my culture. (9)
- myStrength was not in a language that I want to use. (10)
- I didn't have consistent access to a smartphone or internet connection to use myStrength. (11)
- I didn't have enough space on my phone or tablet to use myStrength. (12)
- I wanted to handle the problem myself. (13)
- I felt like I no longer needed myStrength. (14)
- I felt like I had reached my goals using myStrength. (15)
- I was using other technologies to support my mental health that work better for me than myStrength. (16)
- I was using other strategies to support my mental health that work better for me than myStrength. (17)
- I just wanted to try myStrength out. (18)
- I wanted to use only traditional mental health services. (19)
- Other (Please explain): (20) ________________________________________________

Display This Question:

If During the period that you were using myStrength for ${q://QID127/ChoiceGroup/SelectedChoices}, h... = Yes

Q28 How long ago did you stop using myStrength? (Select one answer.)

- About 2 months ago (1)
- About 1 month ago (2)
- About 2 to 3 weeks ago (3)
- About 1 week ago (4)
- Less than one week ago (5)

| Page Break |  |
| --- | --- |

Display This Question:

If What best describes your use of myStrength? (Select one answer.) != I never used it

Q29 The next set of statements are going to ask you about your experiences using myStrength in general. Please rate the extent to which you agree or disagree, using a scale from **Strongly disagree (1)** to **Strongly agree (5)**.

|  | Strongly disagree-1 (1) | Somewhat disagree-2 (2) | Neither agree nor disagree-3 (3) | Somewhat agree-4 (4) | Strongly agree-5 (5) |
| --- | --- | --- | --- | --- | --- |
| I would recommend myStrength to someone like myself. (1) |  |  |  |  |  |
| I find myStrength useful in my daily life. (3) |  |  |  |  |  |
| myStrength meets my mental health needs. (4) |  |  |  |  |  |
| I would not have been able to use myStrength without the technology training. (6) |  |  |  |  |  |
| I think myStrength is easy to use. (7) |  |  |  |  |  |
| Using myStrength improves my mental health. (8) |  |  |  |  |  |
| Because I used myStrength, I know how to deal with feeling lonely. (9) |  |  |  |  |  |
| Because I used myStrength, I am more likely to reach out for help. (11) |  |  |  |  |  |
| Using myStrength makes me feel connected to other people. (12) |  |  |  |  |  |
| Using myStrength has helped me get access to support sooner than I would have if I did not use it. (13) |  |  |  |  |  |
| Using myStrength has helped me detect symptoms related to my mental health. (14) |  |  |  |  |  |
| I feel that as a result of my using myStrength, others know about me more than I am comfortable with. (15) |  |  |  |  |  |
| It is easy to fit myStrength into my everyday life and activities. (16) |  |  |  |  |  |
| I have the resources necessary to use myStrength. (18) |  |  |  |  |  |
| I can get help from others when I have difficulties using myStrength. (19) |  |  |  |  |  |

| Page Break |  |
| --- | --- |

Display This Question:

If What best describes your use of myStrength? (Select one answer.) != I never used it

Q30 The next set of statements are going to ask your thoughts on how culturally sensitive myStrength is. Please rate the extent to which you agree or disagree, using a scale from **Strongly disagree (1)** to **Strongly agree (5)**.

|  | Strongly disagree-1 (1) | Somewhat disagree-2 (2) | Neither agree nor disagree-3 (3) | Somewhat agree-4 (4) | Strongly agree-5 (5) |
| --- | --- | --- | --- | --- | --- |
| myStrength values and respects cultural differences. (1) |  |  |  |  |  |
| myStrength demonstrates knowledge about my culture. (2) |  |  |  |  |  |

| Page Break |  |
| --- | --- |

Q32 **The next part of the survey is going to ask about your well-being and health.**

Q33 The next set of statements are going to ask you about how you have been feeling during the past 30 days. For each statement, please rate how often you have been feeling that way over the past 30 days, on a scale from **None of the time (1)** to **All of the time (5).**
   
During the last 30 days, about how often did you feel...

|  | None of the time-1 (1) | A little of the time-2 (2) | Some of the time-3 (3) | Most of the time-4 (4) | All of the time-5 (5) |
| --- | --- | --- | --- | --- | --- |
| ...tired out for no good reason? (1) |  |  |  |  |  |
| ...nervous? (2) |  |  |  |  |  |
| ...so nervous that nothing could calm you down? (3) |  |  |  |  |  |
| ...hopeless? (4) |  |  |  |  |  |
| ...restless or fidgety? (5) |  |  |  |  |  |
| ...so restless you could not sit still? (6) |  |  |  |  |  |
| ...depressed? (7) |  |  |  |  |  |
| ...that everything was an effort? (8) |  |  |  |  |  |
| ...so sad that nothing could cheer you up? (9) |  |  |  |  |  |
| ...worthless? (10) |  |  |  |  |  |

Q34
Many people have experienced (or are currently experiencing) a mental health challenge that impacts their ability to function in their family, relationships, or workplace.  Many different terms are used to refer to those experiences—some terms include “mental illness”, “mental health challenge”, “emotional distress,” “psychological disorder,” “mental health condition,” etc. 


Have you or do experience mental health challenges or concerns? (Select one.)

- Yes, I have been diagnosed with a mental health condition (1)
- Yes, I experience mental health challenges but have not been diagnosed by a professional (2)
- No, I don't experience mental health concerns (3)
- Other (Please explain): (4) ________________________________________________
- I prefer not to answer. (5)

| Page Break |  |
| --- | --- |

Display This Question:

If Many people have experienced (or are currently experiencing) a mental health challenge that impac... = Yes, I have been diagnosed with a mental health condition

Or Many people have experienced (or are currently experiencing) a mental health challenge that impac... = Yes, I experience mental health challenges but have not been diagnosed by a professional

Q36
The next set of questions are about mental health stigma. Stigma can cause people to feel badly for something that is out of their control. Keep in mind, these statements don’t represent our views of mental illness. 


Throughout this section, the term “mental illness” will be used. However, there are other terms that could be used, such as mental health challenge, psychological disorder, or mental health condition.

Please rate the extent to which you agree or disagree, using a scale from **Strongly disagree (1)** to **Strongly agree (5)**.

|  | Strongly disagree-1 (1) | Somewhat disagree-2 (2) | Neither agree nor disagree-3 (3) | Somewhat agree-4 (4) | Strongly agree-5 (5) |
| --- | --- | --- | --- | --- | --- |
| Having a mental illness has spoiled my life (2) |  |  |  |  |  |
| I am embarrassed or ashamed that I have a mental illness (4) |  |  |  |  |  |
| I feel inferior to others who don’t have a mental illness (6) |  |  |  |  |  |
| I don’t talk about myself much because I don’t want to burden others with my mental illness (7) |  |  |  |  |  |
| Being around people who don’t have a mental illness makes me feel out of place or inadequate (11) |  |  |  |  |  |
| I avoid getting close to people who don’t have a mental illness to avoid rejection (12) |  |  |  |  |  |
| My symptoms interfere less and less with my life (14) |  |  |  |  |  |
| My symptoms seem to be a problem for shorter periods of time each time they occur (15) |  |  |  |  |  |

| Page Break |  |
| --- | --- |

Q37 Please rate the extent to which you agree or disagree, using a scale from **Strongly disagree (1)** to **Strongly agree (5)**.

|  | Strongly disagree-1 (1) | Somewhat disagree-2 (2) | Neither agree nor disagree-3 (3) | Somewhat agree-4 (4) | Strongly agree-5 (5) |
| --- | --- | --- | --- | --- | --- |
| I know when to ask for help (1) |  |  |  |  |  |
| I am willing to ask for help (2) |  |  |  |  |  |
| I ask for help when I need it (3) |  |  |  |  |  |

End of Block: Well-Being and Health

Start of Block: Connectedness, Loneliness, and Isolation

Q38 **The next set of questions ask about your feelings of connectedness, loneliness, and isolation.**   People may feel lonely or isolated even if they have people in their lives. For many, the pandemic has worsened feelings of loneliness because of the changes to our everyday lives. Some people may not be able to participate in typical in-person activities.

Q39 The next questions are about how you feel about different aspects of your life. For each one, please answer how often you feel that way.

Q40 First, how often do you feel that you lack companionship? Is it…

- Hardly ever (1)
- Some of the time (2)
- Often (3)

Q41 How often do you feel left out? Is it...

- Hardly ever (1)
- Some of the time (2)
- Often (3)

Q42 How often do you feel isolated from others? Is it...

- Hardly ever (1)
- Some of the time (2)
- Often (3)

| Page Break |  |
| --- | --- |

Q43 The following statements describe how people sometimes feel. For each statement, please indicate how often you feel the way described by selecting a number in the space provided, on a scale from **Never (1)** to **Always (4)**. Here is an example:  

 How often do you feel happy?   If you never felt happy, you would respond "never"; if you always feel happy, you would respond "always!'    

 How often do you feel...

|  | Never-1 (1) | Rarely-2 (2) | Sometimes-3 (3) | Always-4 (4) |
| --- | --- | --- | --- | --- |
| ...that you are "in tune" with the people around you? (1) |  |  |  |  |
| ...that you lack companionship? (2) |  |  |  |  |
| ...that there is no one you can turn to? (3) |  |  |  |  |
| ...alone? (4) |  |  |  |  |
| ...part of a group of friends? (5) |  |  |  |  |
| ...that you have a lot in common with the people around you? (6) |  |  |  |  |
| ...that you are no longer close to anyone? (7) |  |  |  |  |
| ...that your interests and ideas are not shared by those around you? (8) |  |  |  |  |
| ...outgoing and friendly? (9) |  |  |  |  |
| ...close to people? (10) |  |  |  |  |
| ...left out? (11) |  |  |  |  |
| ...that your relationships with others are not meaningful? (12) |  |  |  |  |
| ...that no one really knows you well? (13) |  |  |  |  |
| ...isolated from others? (14) |  |  |  |  |
| ...you can find companionship when you want ii? (15) |  |  |  |  |
| ...that there are people who really understand you? (16) |  |  |  |  |
| ...shy? (17) |  |  |  |  |
| ...that people are around you but not with you? (18) |  |  |  |  |
| ...that there are people you can talk to? (19) |  |  |  |  |
| ...that there are people you can turn to? (20) |  |  |  |  |

| Page Break |  |
| --- | --- |

Q44 Please select the number that best describes how many persons in your local area you feel you can depend on or feel close to.


How many persons in your local area do you feel like you can depend on or feel close to?

- None (1)
- 1 to 2 (2)
- More than 2 people (3)

Q45 The next questions are about interactions with others. For each question, please rate how much (or how often) you have participated in these activities during the past week. Unless specified otherwise, the activity can be in-person, by telephone, or online.

|  | None (1) | 1 to 2 times per week (2) | 3 to 6 times per week (3) | Daily (4) |
| --- | --- | --- | --- | --- |
| In the past week, about how often did you go to meetings of clubs, religious meetings, or other groups that you belong to? (in-person, by telephone, or online) (1) |  |  |  |  |
| How many times in the past week did you spend time IN PERSON with someone who does not live with you? (2) |  |  |  |  |
| How many times in the past week did you talk with friends or relatives on the TELEPHONE? (3) |  |  |  |  |
| How many times in the past week did you talk with friends or relatives using VIDEO DEVICES (such as FaceTime, Skype)? (4) |  |  |  |  |
| How many times in the past week did you communicate with friends or relatives using the INTERNET (such as e-mail, social media)? (5) |  |  |  |  |

Q46 **The next part of the survey is going to ask about your access to mental healthcare services.**

Q49 **In the past 2 months**, have you visited an Emergency Department (ED) for problems related to your mental health, emotions, or nerves? (Select one.)

- Yes (1)
- No (2)
- I don’t know. (3)
- I prefer not to answer. (4)

Q50 **In the past 2 months**, have you been hospitalized for problems related to your mental health, emotions, or nerves? (Select one.)

- Yes (1)
- No (2)
- I don’t know. (3)
- I prefer not to answer. (4)

Q51 **In the past 2 months**, how frequently have you used professional health services (psychiatrist, psychologist, clinical social worker, etc.) for problems related to your mental health, emotions, or nerves? This can refer to an in-person visit or remote contact such as a phone call or videocall. (Select one.)

- Daily (1)
- Weekly (2)
- Monthly (3)
- A few times a year (4)
- Never (5)
- I don’t know. (6)
- I prefer not to answer. (7)

End of Block: Mental Healthcare Services

Start of Block: Demographics

Q52 **The last part of the survey is going to ask about your background and demographics.**   We ask this information to ensure we represent the viewpoints of your community. We ask these questions of all participants. Some may feel personal in nature. Remember, you can choose not to answer questions you don't feel comfortable answering.

Q55 **In the last 2 months**, have you or anyone you know been diagnosed with the coronavirus/COVID-19? (Select one answer.).

- Yes (1)
- No (2)
- I prefer not to answer. (3)

| Page Break |  |
| --- | --- |

Display This Question:

If In the last 2 months, have you or anyone you know been diagnosed with the coronavirus/COVID-19? (... = Yes

Q56 Who has been diagnosed? (Select all that apply.)

- Self (1)
- Partner/spouse (2)
- Child (3)
- Parent (4)
- Grandparent (5)
- Friend (6)
- Colleague (7)
- Acquaintance (8)
- Other (Please explain): (9) ________________________________________________
- I prefer not to answer. (10)

| Page Break |  |
| --- | --- |

Q57 **In the last 2 months**, have you had a change in your employment because of the coronavirus/COVID-19? (Select one answer.)

- Lost my job (1)
- Had my hours reduced (2)
- Same job and hours, but working less due to children at home/other caregiving responsibilities (3)
- Working from home/remotely - my choice (4)
- Working from home/remotely - required by employment/government mandate (5)
- Other (Please explain): (6) ________________________________________________
- No change in my employment (7)
- I prefer not to answer. (8)

| Page Break |  |
| --- | --- |

Q58 What else would you like to share with us?


This includes but is not limited to sharing more information about your thoughts on this program, your mental health or wellness, or coping strategies and resources. If you prefer not to answer, then you can leave this blank.

________________________________________________________________

________________________________________________________________

________________________________________________________________

________________________________________________________________

________________________________________________________________

End of Block: Demographics

Start of Block: Debrief

Q59
You have reached the end of the survey. Thank you so much for your participation! To submit your responses, select "**Submit responses**" at the bottom of this page. 

 Upon survey submission, you will be redirected to a form where you may enter your email address to receive a $10 eGift card for your participation. Your email address will NOT be linked to the information you provided in this survey.

 All information shared will be kept confidential. Findings from the survey will be used to help understand how myStrength can best serve your community. 

 If you have any questions or concerns about the study, please contact *[enter contact information]*.
   
 Please know resources are available to you if you choose to use them.

 **The Suicide Prevention Lifeline**
 They connect callers to trained counselors 24/7, for free. They also provide a chat function on their website. 
 Phone: 1-800-273-8255
 Website: https://suicidepreventionlifeline.org/

 **Crisis Text Line**
 It is free, 24/7 support for those who are feeling low.
 Text 741741 from anywhere in the US to text with a trained Crisis Counselor. 
 Website: https://www.crisistextline.org/

 **Marin County Behavioral Health and Recovery Services Access Line**
 The BHRS Access Line is open 24/7 to answer questions about services, arrange appointments, and make referrals.
 Phone: 1-888-818-1115

End of Block: Debrief

Survey 4

Start of Block: Intro

Hello, my name is [name], and I'm looking for [participant name]. I am calling from the University of California, Irvine. 
  
Last year, you participated in a program with [county staff name] in Marin County called Help@Hand where you were invited to technology training classes and were shown an application called myStrength. We are calling you today to learn more about your experiences with myStrength. These questions should take only 10 minutes of your time and you will receive a $10 electronic gift card for taking the survey. Do you have time to talk with me now?
 
*If yes: [review study information below. The approved study information sheet can be emailed to participants upon request]*
*If no: [ask whether survey can be scheduled for a later time]*

**Study Information**

- Your participation in this research study is voluntary. You may choose to skip any questions or withdraw at any time.
- We would like you to complete a survey to learn more about your experience using the myStrength application.
- Possible risks/discomforts associated with the survey are uneasy feelings related to sharing information about your mental health and wellness.
- You will receive a $10 electronic gift card to a Target or Amazon for your participation in this study.
- All research data collected will be stored securely and confidentially on a secure server. Information will be password protected and maintained in an encrypted format.
- The research team, authorized UCI personnel, and regulatory entities, may have access to your study records to protect your safety and welfare.
- Researchers will use your information to conduct this study. Once the study is done using your information, we may share them with other researchers so they can use them for other studies in the future. We will not share your name or any other private identifiable information that would let the researchers know who you are. We will not ask you for additional permission to share this de-identified information.

Do you have any questions before we begin the survey?

Enter the participant ID below

________________________________________________________________

End of Block: Intro

Start of Block: Current MS use

Last year, you were invited to test an application called myStrength. myStrength is an application that can be used on a computer, tablet, or smartphone and includes tools like mindfulness to build resiliency and balance emotions and stress. myStrength also provides support for common conditions or experiences such as chronic pain, insomnia, depression, anxiety, alcohol and drug use, and trauma.   

Our records show people have stopped using myStrength. We would like to learn more about why people have stopped using myStrength.   

We did not create the Help@Hand project, technology training classes, or the myStrength application. We would really appreciate your opinions and honest feedback. You will only be asked to give feedback on the technology training classes and myStrength -- not your opinion on peers or Promotores. What you share will help Marin County create and implement mental health, wellness, and technology programs for older adults.

Q6 Are you currently using myStrength? (Select one answer.)

- Yes, I currently use myStrength (1) (Skip to Section B: Current Users)
- No, but I did use myStrength in the past (2) (Skip to Section A: Non-Users)
- No, and I never used myStrength (3) (Skip to Section A: Non-Users)

End of Block: Current MS use

Section A (Non-Users)

Q7 I am going to read you a list of examples of technical issues you may have experienced that prevented you from using myStrength. After each statement, let me know if you have experienced that issue.
 
What **technical issues, if any,** did you experience that prevented you from using myStrength?

- Didn't know how or forgot how to use the tablet, smartphone, or computer (1)
- Lost access to myStrength and couldn't figure out how to get it back (e.g., forgot password) (2)
- Didn't have access to a smartphone, tablet, or computer to use myStrength (3)
- Didn't have access to or couldn't pay for an internet/WiFi connection to use myStrength (4)
- Didn't have enough data on your data plan or enough space on your phone or tablet to use myStrength (5)
- What other technical issues, if any, did you experience that prevented you from using myStrength? (6) ________________________________________________
- None (7)

Q8 I am going to read you a list of examples of life events you may have experienced that prevented you from using myStrength. After each statement, let me know if you have experienced that life event. 
 
What **life events, if any,** did you experience that prevented you from using myStrength?

- Experienced an illness or injury (1)
- A close family member or friend experienced an illness or injury or passed away (2)
- Moved (3)
- Changed jobs (4)
- Lost your job (7)
- What other life events, if any, did you experience that prevented you from using myStrength? (5) ________________________________________________
- None (6)

Q9 I am going to read you a list of examples of other reasons that prevented you from using myStrength. After each statement, let me know if that reason applies to you. 
 
What **other reasons, if any,** prevented you from using myStrength?

- Didn't have support from the program, such as staff, nurse interns, or Promotores (1)
- Privacy concerns (2)
- Worried what other people might think of me if they knew I used myStrength (3)
- Incorrect Spanish translations or not enough content in Spanish (4)
- Didn't think myStrength [was/would be] helpful / didn't get enough benefit from myStrength (e.g., content doesn't update enough, content not aligned with cultural or religious beliefs, etc.) (5)
- Not interested in using technology (e.g., smartphone, tablet, computer, or tools like myStrength) (6)
- No time / too busy to use it (7)
- I forgot to use it (8)
- What other reasons, if any, prevented you from using myStrength? (9) ________________________________________________
- None (10)

Q10 Now I'm going to ask you about your opinions of the myStrength application. As a reminder, we did not create the technology training classes or the myStrength application. You will not be asked to provide your opinion on peers or Promotores. We will only ask for your feedback on the technology training classes and myStrength. We would really appreciate your honest feedback.

Q11 On a scale from 1 to 10, with 1 being the lowest score and 10 being the highest, how would you rate myStrength?

- 1 (1)
- 2 (2)
- 3 (3)
- 4 (4)
- 5 (5)
- 6 (6)
- 7 (7)
- 8 (8)
- 9 (9)
- 10 (10)

Q12 Do you intend to use myStrength in the future?

- Yes (1)
- No (2)
- Not sure (3)

Q13 What, if anything, would make it easier for you to use myStrength?

- On-going support provided by staff in Marin County (1)
- More technology training classes (2)
- Free access to internet/WiFi (3)
- Free access to devices, like a smartphone, tablet, or computer (4)
- What other things, if anything, would make it easier for you to use myStrength? (5) ________________________________________________
- None / wouldn't use myStrength (6)

End of Block: Section A (Non-Users); Skip to Section C (for all)

Section B (Current Users)

Q14 You said you are currently using the myStrength application you tested out last year. Is this correct?   

*If yes: continue with questions below
If no: return to previous question*

Q15 Tell me more about how you access myStrength. 
*[Want to probe here because dashboard indicates no one is still using myStrength; could they have been locked out of old account and started a new one? could they be using an account provided by their insurance company? etc.]*

________________________________________________________________

Q16 Why do you continue to use myStrength? For example, what do you like about myStrength? How does it benefit you?

________________________________________________________________

Q17 I am going to read you a list of examples of technical issues you may have experienced that make it difficult to use myStrength. After each statement, let me know if you have experienced that issue. 
 
What **technical issues, if any,** did you experience that make it difficult to use myStrength?

- Didn't know how or forgot how to use the tablet, smartphone, or computer (1)
- Lost access to myStrength and couldn't figure out how to get it back (e.g., forgot password) (2)
- Didn't have access to a smartphone, tablet, or computer to use myStrength (3)
- Didn't have access to or couldn't pay for an internet/WiFi connection to use myStrength (4)
- Didn't have enough data on your data plan or enough space on your phone or tablet to use myStrength (5)
- What other technical issues, if any, did you experience that make it difficult to use myStrength? (6) ________________________________________________
- None (7)

Q18 I am going to read you a list of examples of life events you may have experienced that make it difficult to use myStrength. After each statement, let me know if you have experienced that life event. 
 
What **life events, if any,** did you experience that make it difficult to use myStrength?

- Experienced an illness or injury (1)
- A close family member or friend experienced an illness or injury or passed away (2)
- Moved (3)
- Changed jobs (4)
- Lost your job (7)
- What other life events, if any, did you experience that make it difficult to use myStrength? (5) ________________________________________________
- None (6)

Q19 I am going to read you a list of examples of other reasons that make it difficult to use myStrength. After each statement, let me know if that reason applies to you. 
 
What **other reasons, if any,** make it difficult to use myStrength?

- Didn't have support from the program, such as staff, nurse interns, or Promotores (1)
- Privacy concerns (2)
- Worried what other people might think of you if they knew you used myStrength (3)
- Incorrect Spanish translations or not enough content in Spanish (4)
- Didn't think myStrength [was/would be] helpful / didn't get enough benefit from myStrength (e.g., content doesn't update enough, content not aligned with cultural or religious beliefs, etc.) (5)
- Not interested in using technology (e.g., smartphone, tablet, computer, or tools like myStrength) (6)
- No time / too busy to use it (7)
- I forgot to use it (8)
- What other reasons, if any, make it difficult to use myStrength? (9) ________________________________________________
- None (10)

Q20 Now I'm going to ask you about your opinions of the myStrength application. As a reminder, we did not create the technology training classes or the myStrength application. You will not be asked to provide your opinion on peers or Promotores. We will only ask for your feedback on the technology training classes and myStrength. We would really appreciate your honest feedback.

Q21 On a scale from 1 to 10, with 1 being the lowest score and 10 being the highest, how would you rate myStrength?

- 1 (1)
- 2 (2)
- 3 (3)
- 4 (4)
- 5 (5)
- 6 (6)
- 7 (7)
- 8 (8)
- 9 (9)
- 10 (10)

Q22 Do you intend to use myStrength in the future?

- Yes (1)
- No (2)
- Not sure (3)

Q23 What, if anything, would make it easier for you to use myStrength?

- On-going support provided by staff in Marin County (1)
- More technology training classes (2)
- Free access to internet/WiFi (3)
- Free access to devices, like a smartphone, tablet, or computer (4)
- What other things, if anything, would make it easier for you to use myStrength? (5) ________________________________________________
- None (6)

End of Block: Section B (Current Users); Skip to Section C (For all)

Section C (for all)

We're almost done. Thank you so far for sharing your feedback with me. For the last part, I'm going to ask you a few more questions about how the technology training and myStrength application did or did not affect you. Remember, we are only asking about the technology training classes and myStrength not peers or Promotores. 


As a reminder, the technology training were the classes you were invited to to learn about how to use devices and the internet. myStrength is an application that can be used on a computer, tablet, or smartphone and includes tools like mindfulness to build resiliency, balance emotions and stress, and track things like sleep.

Q25 What **things, if any, do you do with the computer, tablet, smartphone or the internet** to support your mental health and wellness because you participated in the technology training classes or myStrength application?


For example, you might watch videos online or use video calling to talk with a therapist because of something you learned during the technology training or by using myStrength. 


It's also okay if your participation in the program didn't affect how you use the computer, tablet, or smartphone to support your mental health and wellness.

________________________________________________________________

________________________________________________________________

________________________________________________________________

________________________________________________________________

________________________________________________________________

Q27
*Possible Probe:* *How, if at all, has the technology training classes or myStrength application influenced the things you do on the computer, tablet, or smartphone to support your mental health and wellness?*

________________________________________________________________

Q28
*Possible Probe:* *What have you learned about technology, if anything, because you participated in the Help@Hand program, technology training, or myStrength application?*

________________________________________________________________

Q29
*Possible Probe:* *What have you learned about mental health, if anything, because you participated in the Help@Hand program and myStrength application?*

________________________________________________________________

Q30 What else you'd like to share with us?

________________________________________________________________

Q31 We have reached the end of the survey. Thank you so much for your participation! We can offer a $10 Target or Amazon gift card, which will be sent to your email address. Which one would you like?

- Target (1)
- Amazon (2)

Q33 This is the email address that we have on file for you *[share email address]*. Is this still a good email to send the gift card? 
 
*[if not, write down updated email address]*
 
You will receive your gift card by email within a week. Do you have any questions for me? 
 
Thank you again for your time. Have a nice day.

End of Block: Section C (for all)

Interview guide

**1. Interview guide for participants who stop participating during technology training**

**Introduction**

*Hi, [participant name]. My name is [name], and I'm calling from the University of California, Irvine because you are part of Marin County's Technology Training Tech4Life and myStrength program. Marin County would love to learn about your experience with this program. Your feedback will help improve the program for community members like you.*

*According to our records, you decided to stop participating in the program [verify with participant]. We would like to ask you if you would be willing to take part in an interview, which will take about 30 minutes. Participation in this interview is voluntary, and you may decide to stop participating at any point. You will receive a $20 gift card for taking part in the interview.*

*We are interested in hearing about your experiences with the Help@Hand and Technology Training Program, including what worked well or didn’t work well. We will not ask about your personal history, and you should only share what you feel comfortable with. We will be summarizing the main themes from our interviews in a summary report. Your name will not be included in any report or linked to any of your comments.*

*We’d like to audio record our interview for analysis. Would it be okay if I audio recorded our conversation?*

*[If yes, record and state]: It’s [date, time] and I’m with participant #X, who has agreed to be audio recorded.*

*[If no, take notes]*

*What questions do you have before we begin?*

| **Topic** | **Construct** | **Question** | **Notes** |
| --- | --- | --- | --- |
| Rapport building / warm-up | N/A | Before we begin, I’d like to learn more about you. Tell me a little bit about yourself...   - (e.g., something you like to do for fun, if you have any pets, etc.) |  |
| Initial Impressions and Experience | Awareness | How did you become aware of the Marin County Technology Training and myStrength program? |  |
|  | Motivation | Why did you decide to participate in the Marin County Technology Training and myStrength program? |  |
|  | Expectations | What were your expectations of the Marin County Technology Training and myStrength program?  Did the program meet your expectations?   - Why / why not? |  |
| Overall Experience of PROGRAM | General perception | In general, what was your experience like with the Marin County Technology Training and myStrength program?  How easy was it to sign up for the Marin County Technology Training and myStrength program? |  |
|  | Benefits | What parts of the Marin County Technology Training and myStrength program were beneficial to you, if any?  How were they beneficial to you? |  |
|  | Improvements | What parts of the Marin County Technology Training and myStrength program were not beneficial to you, if any?  Why weren't these beneficial?  How would you change these to better fit your needs? |  |
|  | Challenges | Did you experience any challenges with the Marin County Technology Training and myStrength program?  If yes: What challenges did you experience?  Is there anything you would change about the program that would/could prevent these challenges? |  |
|  | Loneliness, isolation, and connection | For many, the pandemic has worsened feelings of loneliness and isolation because of the changes to our everyday lives. Some people may not be able to participate in typical in-person activities.  How often do you feel isolated or lonely?  How did the Marin County Technology Training and myStrength program impact your feelings of connection with people, if at all? |  |
| Outcomes | Loneliness | The next questions are about how you feel about different aspects of your life. For each one, please answer how often you feel that way.    First, how often do you feel that you lack companionship? Is it…   - Hardly ever - Some of the time - Often     How often do you feel left out? Is it...   - Hardly ever - Some of the time - Often     How often do you feel isolated from others? Is it...   - Hardly ever - Some of the time - Often |  |
|  | Stigma | Please rate the extent to which you agree or disagree, using a scale from **Strongly disagree (1)** to **Strongly agree (5)**.  My self-esteem would increase if I talked to a therapist.   - Strongly disagree-1 - Somewhat disagree-2 - Neither agree nor disagree-3 - Somewhat agree-4 - Strongly agree-5 |  |
|  | Healthcare utilization/access | In the past 2 months, how frequently have you used professional health services (psychiatrist, psychologist, clinical social worker, etc.) for problems related to your mental health, emotions, or nerves? This can refer to an in-person visit or remote contact such as a phone call or videocall. (Select one answer.)   - Daily - Weekly - Monthly - A few times a year - Never - I don’t know. - I prefer not to answer. |  |
| Experience with County Staff and Peers |  | In general, what was your experience with the County staff you interacted with as part of the Marin County Technology Training and myStrength program?   - *Possible probe around different roles (e.g., Lorraine and Damaris?)*   What type of help or support did they provide? |  |
| Experience with Nurse Interns |  | (*if participant received assistance from a nurse intern*)  As part of the Marin County Technology Training and myStrength program, you received help from a nurse intern.  (if available): Your nurse intern was [NAME].  How helpful was the nurse intern in supporting you to participate in the program?  In general, what was your experience like with the nurse intern?  How often did the nurse intern help you?  What types of help or support did the nurse intern provide? |  |
| Experience with Promotores |  | (*if participant received assistance from promotores)*  As part of the Marin County Technology Training and myStrength program, you received help from promotores.  (if available): Your promotora was [NAME].  How helpful as the promotora in supporting you to participate in the program?  In general, what was your experience like with the promotora?  How often did the promotora help you?  What types of help or support did the promotora provide? |  |
| Attrition in the Program | Timing of pilot drop-out | When did you stop participating in the Marin County Technology Training and myStrength program? |  |
|  | Reasons for drop-out | Why did you stop participating in the Marin County Technology Training and myStrength program?  What would have made you more likely to continue participating in in the Marin County Technology Training and myStrength program? |  |
|  | Experience and perception of technology training | Which technology training sessions did you participate in, if any?   - How did they participate? Live, video?     If participated in any of the trainings:   - How was the training? - What things did you learn that you didn't know before participating? - How do you think the training will affect your ability to use technology to support your well-being? - How easy was it to sign up for/take part in the training? |  |
|  | Likeliness of participating in similar programs | How likely is it that you would take part in a similar program again in the future, if available to you? |  |
| Other Ways to Support Well-being and Mental Health | Current and future use of digital tech to support mental health and well-being | What resources and strategies, if any, do you use to support your well-being (and/or mental health)?  How useful are these resources in supporting your well-being?  Would you be interested in using technology in the future to support your mental health and well-being? (give examples)   - Why / why not? |  |
| Other | N/A | What else would you like to share or talk about? |  |

**Closing Script**

*We’ve reached the end of the interview. Thank you for your participation! Your feedback will help improve the program for older adults in Marin County. We genuinely appreciate the time you took to speak with me today. Your electronic gift card will be sent to your email address. Your email address will not be linked to the information you provided in this interview. All information shared will be kept confidential.*

*If you have any follow up comments or questions, please feel free to contact us at [enter contact information]. Thank you again and have a great day!*

**2. Interview guide for participants who complete program**

**Introduction**

*Hi, [participant name]. My name is [name], and I'm calling from the University of California, Irvine because you are part of Marin County's Technology Training Tech4Life and myStrength program. Marin County would love to learn about your experience with this program and myStrength. Your feedback will help improve the program for community members like you.*

*We are interested in hearing about your experiences with the Technology Training Program and myStrength, including what worked well or didn’t work well. The interview will take about 1 hour. You will receive a $20 gift card for taking part in the interview. Participation in this interview is voluntary, and you may decide to stop participating at any point.*

*We will not ask about your personal history, and you should only share what you feel comfortable with. We will be summarizing the main themes from our interviews in a summary report. Your name will not be included in any report or linked to any of your comments.*

*We’d like to audio record our interview for analysis. Would it be okay if I audio recorded our conversation?*

*[If yes, record and state]: It’s [date, time] and I’m with participate #X, who has agreed to be audio recorded.*

*[If no, take notes]*

*What questions do you have before we begin?*

*PRIORITY QUESTIONS ARE IN BLUE TEXT BELOW*

| **Topic** | **Construct** | **Question** | **Notes** |
| --- | --- | --- | --- |
| Rapport building / warm-up | N/A | Before we begin, I’d like to learn more about you. Tell me a little bit about yourself...   - (e.g., something you like to do for fun, if you have any pets, etc.) |  |
| Initial Impressions and Experience | Awareness | How did you become aware of the Marin County Technology Training and myStrength program? |  |
|  | Motivation | Why did you decide to participate in the Marin County Technology Training and myStrength program? |  |
|  | Expectations | What were your expectations of the Marin County Technology Training and myStrength program?  Did the program meet your expectations?   - Why / why not? |  |
| Overall Experience | General perception | In general, what was your experience like with the Marin County Technology Training and myStrength program?  How easy was it to sign up for the Marin County Technology Training and myStrength program? |  |
|  | Benefits | What parts of the Marin County Technology Training and myStrength program were beneficial to you?  How were they beneficial to you? |  |
|  | Improvements | What parts of the Marin County Technology Training and myStrength program were not beneficial to you?  Why weren't these beneficial?  How would you change these to better fit your needs? |  |
|  | Challenges | Did you experience any challenges with the Marin County Technology Training and myStrength program?  If yes: What challenges did you experience?  Is there anything you would change about the program that would/could prevent these challenges? |  |
|  | Loneliness, isolation, and connection | For many, the pandemic has worsened feelings of loneliness and isolation because of the changes to our everyday lives. Some people may not be able to participate in typical in-person activities.  How did the Marin County Technology Training and myStrength program impact your feelings of connection with other people, if at all? |  |
| Outcomes | Loneliness (outcomes) | The next questions are about how you feel about different aspects of your life. For each one, please answer how often you feel that way.    First, how often do you feel that you lack companionship? Is it…   - Hardly ever - Some of the time - Often     How often do you feel left out? Is it...   - Hardly ever - Some of the time - Often     How often do you feel isolated from others? Is it...   - Hardly ever - Some of the time - Often |  |
|  | Stigma | Please rate the extent to which you agree or disagree, using a scale from **Strongly disagree (1)** to **Strongly agree (5)**.  My self-esteem would increase if I talked to a therapist.   - Strongly disagree-1 - Somewhat disagree-2 - Neither agree nor disagree-3 - Somewhat agree-4 - Strongly agree-5 |  |
|  | Healthcare utilization / access | In the past 2 months, how frequently have you used professional health services (psychiatrist, psychologist, clinical social worker, etc.) for problems related to your mental health, emotions, or nerves? This can refer to an in-person visit or remote contact such as a phone call or videocall. (Select one answer.)   - Daily - Weekly - Monthly - A few times a year - Never - I don’t know. - I prefer not to answer. |  |
| Experience with County Staff and Peers |  | In general, what was your experience with the County staff you interacted with as part of the Marin County Technology Training and myStrength program?   - *Possible probe around different roles (e.g., Lorraine, Damaris)*   What type of help or support did they provide? |  |
| Experience with Nurse Interns |  | **(*if participant received assistance from nurse interns*)**  As part of the Marin County Technology Training and myStrength program, you received help from a nurse intern.  How helpful was the nurse intern in supporting you to participate in the program?  In general, what was your experience like with the nurse intern?  How often did the nurse intern help you?  What types of help or support did the nurse intern provide? |  |
| Experience with Promotores |  | **(*if participant received assistance from promotores)***  As part of the Marin County Technology Training and myStrength program, you received help from promotores.  (if available): Your promotora was [NAME].  How helpful as the promotora in supporting you to participate in the program?  In general, what was your experience like with the promotora?  How often did the promotora help you?  What types of help or support did the promotora provide? |  |
| Technology Training | Technology training perception and experience | Which technology training sessions did you participate in, if any?   - How did they participate? Live, video?     If participated in any of the trainings:   - How was the training? - What things did you learn that you didn't know before participating? - How do you think the training will affect your ability to use technology to support your well-being? - How easy was it to sign up for/take part in the training?     If participated in none or not all: Why didn't you participate? (or Why did you not participate in all the trainings?) |  |
| myStrength Experience | myStrength expectations | What were you hoping to get out of using myStrength?  Did it succeed in providing that to you / meeting your expectations? |  |
|  | Getting started | How was it getting started with myStrength? |  |
|  | Frequency of use | How often did you use myStrength?  Are you still using myStrength?  If yes to still using: Have you ever taken a break from using myStrength? If so, why?  If no to still using:   - Why did you stop using myStrength?   *Possible probes:*   - - Did you find that it took too much of your time or was too difficult to use?   - Did you have any concerns about sharing your mental health information in myStrength?   - Did you have any concerns about other people knowing that you were using myStrength?   - Do you feel like you got what you wanted to out of the app?   - Did something happen in your life which made myStrength less useful? - People have a range of perspectives on apps after they stop using them. Some people feel like the app wasn’t very important to them, others feel guilty about stopping to use something which helped them, others continue to use what the app taught them. How do you feel about your time using myStrength? Why do you feel that way? - What would you change to make it more likely you'd use myStrength, if anything? - Do you intend to use myStrength in the future? Why/why not? |  |
| ***If any use of myStrength:*** | | | |
|  | Acceptability [Likeability] | What did you like about myStrength? Why did you like these?  What did you not like about myStrength? Why didn't you like these? |  |
|  | General experience | In general, what has your experience with myStrength been like?  Can you describe a positive experience you had / a time when you felt particularly happy when using myStrength?  Can you describe a negative experience / a time when you felt negative emotions when using myStrength? |  |
|  | Lifestyle fit and typical use | Describe a typical day when you use(d) myStrength, including when you use it and what you do on the app. |  |
|  | Perceived Usefulness | How useful was myStrength for you? Why?  For many, the pandemic has worsened feelings of loneliness and isolation because of the changes to our everyday lives. For some, the pandemic may have made it more difficult to access in-person mental health services.  Do you feel myStrength was more/less helpful/needed as a resource during the pandemic than it would have been otherwise? Why?  Has myStrength changed how you manage your mental health / well-being? If so, how? |  |
|  | Appropriateness | Did myStrength meet your mental health and well-being needs? If so, how? If not, why not?  How effective do you think myStrength has been in providing you with the support you need? |  |
|  | Loneliness, Isolation, and Connection | How did the myStrength app impact your feelings of connection or loneliness, if at all? |  |
|  | Usability | How easy or difficult was it to use myStrength? |  |
|  | Culture and Cultural Competency | What role has your upbringing or culture played in how you think about mental health?  Did you feel myStrength was sensitive/appropriate to your culture? Why / why not?  In general, do you feel that myStrength values and respects cultural differences? Why / why not? |  |
| ***For Spanish Speakers*** | Spanish Translation and Workflow | How easy or difficult was it change the language in myStrength to Spanish?  Tell me about the Spanish language translation.   - Were there any issues with the translation? If so, what were they? - How would you improve the translation?   Was there enough content in Spanish?   - Did you watch any videos in English with Spanish subtitles? What was this like? OR Why not? - What other content in Spanish would you like to see on myStrength? |  |
|  | Privacy | Have you thought about privacy when using myStrength?  Are you concerned that your data may not be private when you use myStrength, e.g., that others may see your data? |  |
|  | Specific feature use and perceptions | What particular features did you like or dislike?  Are there any additional features you felt were missing, that you wished myStrength had? |  |
|  | Likeliness of accessing services in the future | How likely are you to access mental health services in the future, if available to you?   - How did myStrength play a role, if at all? |  |
|  | Perceptions of mental health | Has using myStrength changed or not changed your attitude about mental health generally? If so, how? |  |
|  | Ability to recognize symptoms | Has myStrength helped you in detecting symptoms related to your mental health? If so, how? |  |
| ***Anyone:*** | | | |
| Other Ways to Support Well-being and Mental Health | Current and future use of digital tech to support mental health and well-being | What resources and strategies, if any, do you use to support your well-being (and/or mental health)?  How useful are these resources in supporting your well-being?  How interested would you be in using technology in the future to support your mental health and well-being? |  |
| Other | N/A | What else would you like to share or talk about? |  |

**Closing Script**

*We’ve reached the end of the interview. Thank you for your participation! Your feedback will help improve the program for older adults in Marin County. We genuinely appreciate the time you took to speak with me today. Would you like to receive a $20 electronic gift card for your participation in this interview? We can provide a $20 Target or Amazon eGift card. [if yes, note what kind of gift card the participant wants]. Your electronic gift card will be sent to your email address. Your email address will not be linked to the information you provided in this interview. All information shared will be kept confidential.*

*If you have any follow up comments or questions, please feel free to contact us at [enter contact information]. Thank you again and have a great day!*
