# Supplementary material for: Understanding the Role of Support in Digital Mental Health Programs With Older Adults: Users’ Perspective and Mixed Methods Study
Source: JMIR Form Res. 2022 Dec 13;6(12):e43192. doi: 10.2196/43192 (PMC9795392; doi:10.2196/43192)
Supplement: Multimedia Appendix 2 [file formative_v6i12e43192_app2.docx]

# **Participant Screener**

| **Marin Help@Hand Screener Form**  *(Designed to be webform for high digital literacy participants and by phone for lower digital literacy participants)* | |
| --- | --- |
| What language do you prefer to read this information / [Spanish translation] | - English / [Spanish translation] - Spanish / [Spanish translation] |
| *If English, show English version*  *If Spanish, show Spanish version* | |
| **Overview** | In 2022, Marin County will implement a program called Help@Hand for older adults ages 60+. The project is designed to support older adults in their emotional wellness by providing access to an evidence-based self-help resource for emotional health and overall well-being, as well as support learning technology safely for those who need it. The self-help technology being offered is called myStrength™ and it is designed to help manage distress from life events and challenges. |
| **What is myStrength™?** | myStrength™ is a technology that can be used on a computer, tablet, or smartphone and includes tools like mindfulness to build resiliency and balance emotions and stress. In addition, myStrength™ provides support for common conditions or experiences such as chronic pain, insomnia, alcohol and drug use, opioid and nicotine use, depression, anxiety, and trauma. myStrength™ provides individual assessment and delivers a personalized plan with priority focus areas and reminders to stay on track. |
| **Has** **myStrength™ been explored in Marin County before?** | The Help@Hand project and myStrength™ technology were extensively tested with older adults in Marin county in 2021 and participation in the project was shown to significantly increase confidence in using technology to access health and mental health resources, as well as to reduce loneliness and isolation. |
| **If I want to participate, what is expected of me?** | Participants in Help@Hand will be expected to:   - Be 60 years old or older - Speak, read, and write in either Spanish or English - Establish a myStrength™ account and explore emotional health and well-being supports available - Sign participation agreements - Participate in surveys designed to help Marin learn more about how myStrength™ supports well-being   - (As part of the program, you will be asked to take online surveys that will be sent to your email address. If you don't have an email address, you may be asked to create one. If needed, you will learn about how to create an email address during digital literacy training classes.) - If needed, participate in basic digital literacy training classes, and establish an email account - Following COVID-19 Protocols as established by Help@Hand |
| **What is the purpose of this screener?** | The screener you are about to begin will be used for the purpose of determining your eligibility for this program. If you agree to participate in this screening, you will be asked for information about your medical history. The screener will take approximately 15 minutes to complete. You may complete this online or by phone.  Your participation in this online screening is entirely voluntary. You can choose not to answer questions, or you may stop the questionnaire at any time. **Your information will be used by Help@Hand staff and researchers at the University of California, Irvine to determine eligibility for the program.** |
| **How do I know if I'm eligible for this program?** | If you qualify for the program, Help@Hand staff will call you. If you have any questions, please contact Help@Hand staff member:  Name and contact information |
| **Once you consent to participate in this screener, the survey questions will be visible to you only. Do you agree to participate?** | - Yes - No   Submit  *If no, then send message automatically online or say via phone:* *You answered that you do not agree to participate. Thank you for your time!* *[then exit survey]*  *If yes, proceed to questions:* |
| ***Asked of all potential participants:*** | |
| 1) How old are you? | _____________  *If under 60, then send message automatically online or say via phone: Thank you for your interest! I'm sorry, but based on your responses to the screener, you are not eligible for this program at this time.* |
| 2) Language you prefer to participate in the program: | - English - Spanish - Either English or Spanish - Neither English nor Spanish   *If neither English nor Spanish, then send message automatically online or say via phone: I'm sorry, but the program is currently being offered in only Spanish and English. We thank you for your time and interest.* |
| 3) Do you know how to read and write? | - - Yes   - No   *If No, then send message automatically online or say via phone: Thank you for your interest! I'm sorry, but based on your responses to the screener, you are not eligible for this program at this time.* |
| 4) Do you experience any of the following that might limit your participation in the program? | - Do you have any **physical challenges or impairments**, including but not limited to hearing or vision loss, that would prevent you from being able to use a computer/tablet?   - Yes: (please describe): _______________   - No - Do you have any **mental challenges or impairments** that would prevent you from being able to use a computer/tablet?   - Yes: (please describe): _______________   - No   *If yes to any of the above for Q4 and this is taken online, then someone in Marin County should call potential participant to get more information about their responses to determine eligibility. If yes to any of the above for Q4 and taken via phone, then someone should make sure they explain the impairments and have a protocol to determine if they are eligible or not.* |
| **Digital Literacy** | |
| 5) How comfortable are you using the following devices: | - Laptop or desktop computer:   - Very comfortable   - Somewhat comfortable   - Not at all comfortable - Tablet (e.g., iPad, Samsung Galaxy Tab, etc.):   - Very comfortable   - Somewhat comfortable   - Not at all comfortable - Smartphone (e.g., iPhone, Samsung Galaxy, etc.):   - Very comfortable   - Somewhat comfortable   - Not at all comfortable |
| 6) Which of the following devices do you have? (Select all that apply.) | - Laptop or desktop computer   - My own   - Shared or public - Tablet (e.g., iPad, Samsung Galaxy Tab, etc.)   - My own   - Shared or public - Smartphone (e.g., iPhone, Samsung Galaxy, etc.)   - My own   - Shared or public - I don't have any of these. |
| *If "laptop/desktop" is selected:*  6a) How old is your laptop or desktop (in years)? | _________ |
| 6b) What kind of laptop or desktop do you have (what brand, e.g., Dell, Samsung, Apple, other)? | ___________________________________   - I'm not sure |
| *If "tablet" is selected:*  6c) How old is your tablet (in years)? | _________ |
| 6d) What kind of tablet do you have (what brand, e.g., Apple iPad, Samsung Galaxy Tab, other)? | ___________________________________   - I'm not sure. |
| *If "smartphone" is selected:*  6e) How old is your smartphone (in years)? | _________ |
| 6f) What kind of smartphone do you have (what brand, e.g., Apple iPhone, Samsung Galaxy, other)? | ___________________________________   - I'm not sure. |
| 7) Do you have reliable access to the internet and/or Wi-Fi? | - Yes - No - I'm not sure. |
| 8) Do you use e-mail to communicate? | - Yes - No - I'm not sure. |
| 9) Do you know how to create an email address? | - Yes - No - I'm not sure. |
| 10) Do you know how to create a secure password when you create accounts? | - Yes - No - I'm not sure. |
| 11) Zoom is a video platform that allows you to make a call with one or more people using video. How frequently do you use Zoom by yourself, that is, without the support of anyone else? | - Frequently - Sometimes - Never |
| 12) How often do you use the internet to search for information? | - Frequently - Sometimes - Never |
| ***Asked for potentially low digital literacy group only****, triggered if Q5 = somewhat or not at all for either OR if Q6 = no devices OR if 6a, 6c, 6e = over 5 years old OR if 6b, 6d, or 6f = I'm not sure OR if Q7 = no or I'm not sure OR If Q8 = no or I'm not sure OR if Q9 = no or I'm not sure OR if Q10 = no or I'm not sure or if Q11 = sometimes or never OR if Q12 = sometimes or never:* | |
| 13) Based on your answers, you may be asked to participate in digital literacy training. This would mean participating in 8 group classes (1.5 hours each) on how to use a tablet and myStrength™ . Would you be willing to participate in these classes? | - Yes - No |
| 14) Are you able to travel to an on-site, in-person location to participate in these classes? | - Yes, I can travel to classes myself - Yes, I can travel to classes with support from a family member, friend, or caregiver - No, I cannot travel to classes - Other (please explain): _______________ |
| 15) Do you have any time commitments, such as work or standing medical appointments that would impact your ability to participate? | - Yes, I have work (please explain):________________________ - Yes, I have standing medical appointments (please explain):________________________ - Yes, I have other commitments (please explain):________________________   No |
| *If yes to any for Q15:* | Please indicate days/times you are **not** available: ____________________________________________________ |
| 16) Being fully vaccinated against COVID-19 means receiving:   - 2 doses of Moderna/Pfizer-BioNTech AND a booster at least two weeks ago. - OR 1 dose of J+J AND a booster at least 2 weeks ago   Have you been fully vaccinated against COVID-19? | - Yes - No |
| ***Asked of all potential participants:***  **Demographics** | |
| 18) Name (first and last): | ________________ |
| 19) Phone Number: | ________________ |
| 20) Street Address: | ________________ |
| 21) City: | ________________ |
| 22) State/Province: | ________________ |
| 23) Postal/Zip Code | ________________ |
| 24) Sex or gender: | - Female/woman - Male/man - I prefer to self-identify:________ |
| *End of survey message:*  Thank you so much for taking this survey! If you qualify for the program, Help@Hand staff will call you.  If you have any questions, please contact Help@Hand staff member: (name)  --------------------------------- | |
| **ELIGIBILITY SCORING:**  Basic ineligibility:   - If Q1 < 60 OR - If Q2 = Neither English nor Spanish OR - If Q3 = No OR - Q4 [need to determine what constitutes who would be in/out] OR - If not located in Marin County (based on zip code Q23)   If **eligible** based on basic criteria above AND determined to be lower digital literacy:   - Ineligible if Q13, 14, 15, 16, or 17 = No?   ---------------------------------  **PROCEDURE AFTER ELIGIBILITY IS DETERMINED:**  **IF THEY QUALIFY:**   - If they qualify for the program, we contact them and start the enrollment process - Enrollment form - Sign participation agreement (DocuSign) - Complete a google form with classes dates and times   ***IF THEY DON'T QUALIFY (and they haven't yet been told, e.g., via online):***   - Determine next steps   ---------------------------------  **SORTING INTO COHORTS:**   - [Marin County add criteria later based on needs] | |
